# Exploring the Chemical Diversity of Algerian Plants: Three New Pentacyclic Triterpenoids from *Launaea acanthoclada* Roots

**DOI:** 10.3390/molecules23010080

**Published:** 2017-12-30

**Authors:** Nabila Zergainoh, Maria Letizia Ciavatta, Marianna Carbone, Fatma Bitam, Mohamed Cherif Aberkane, Margherita Gavagnin

**Affiliations:** 1 National Research Council (CNR), Institute of Biomolecular Chemistry (ICB), Via Campi Flegrei, 34, 80078 Pozzuoli (Naples), Italy; n.zergainoh@icb.cnr.it (N.Z.); mcarbone@icb.cnr.it (M.C.); mgavagnin@icb.cnr.it (M.G.); 2 Laboratory of Chemistry and Environmental Chemistry (L.C.C.E), Department of Chemistry, Faculty of Sciences of the Matter, University of Batna 1, 05000 Batna, Algeria; aberkanemc@yahoo.fr; 3 Department of Pharmacy, University of Batna 2, Faculty of Medicine, 05000 Batna, Algeria; fatoma597@gmail.com

**Keywords:** *Launaea acanthoclada*, triterpenoids, lupane, bauerane, structural elucidation, NMR

## Abstract

The chemical study of *Launaea acanthoclada* from South-East Algeria led to the isolation of twelve oxygenated terpenoid compounds, including three new pentacyclic triterpenoids **1**–**3** with either lupane or ursane rearranged skeletons. The structure and the stereochemistry of these compounds were established by spectroscopic methods, including NMR techniques. The chemical pattern of *L. acanthoclada* is in accordance with the triterpenoid scenario of the genus *Launaea* embracing to date lupane, oleane, ursane and taraxastane skeletons. However, the carbon frameworks exhibited by new compounds **1**–**3** have never been reported from *Launaea* species.

## 1. Introduction

*Launaea* Cass. is a small genus of the family Asteraceae (tribe Lactuceae) consisting of about 50 species, most of which are adapted to dry, saline and sandy habitats [1]. *Launaea* genus is mainly distributed in the South Mediterranean, Africa and Southwestern Asia and, in particular, is very common in the North African regions [2,3]. *Launaea acanthoclada* Maire (synonym: *Launaea lanifera* Pau) is one of the nine *Launaea* species endemic of North Africa that are present in the flora of Algeria [1,3]. This plant is a yellow flowered perennial herb up to 40 cm high growing in Algerian superior arid steppes [1,2] and in some regions of Morocco as well as in the arid areas of Southeast Spain [4,5]. Traditionally, *Launaea* species have been used in North African popular medicine for the treatment of several diseases, especially those of liver, lungs and stomach, as well as to heal infected wounds [6]. A number of chemical studies have been previously conducted to investigate the composition of the various *Launaea* species with regards mainly to the volatile fraction (essential oils) and phenolic constituents [6] even though studies on the terpenoid content of selected species including *L. pinnatifida*, *L. asplenifolia*, *L. arborescens*, *L. nudicaulis* and *L. residifolia* have been also appeared in the literature [7].

In the course of our ongoing phytochemical studies on Algerian plants [8,9,10] we have investigated the chemistry of Algerian *Launaea acanthoclada* (local names “kebbad” and “cedada”). To the best of our knowledge, only two previous studies describing phenolic [11] and essential oil [12] components have been reported in the literature for this species. In particular, the essential oil fraction was found to be constituted by apocarotenoids, monoterpene and sesquiterpene hydrocarbons [12]. 

**Figure 1:**
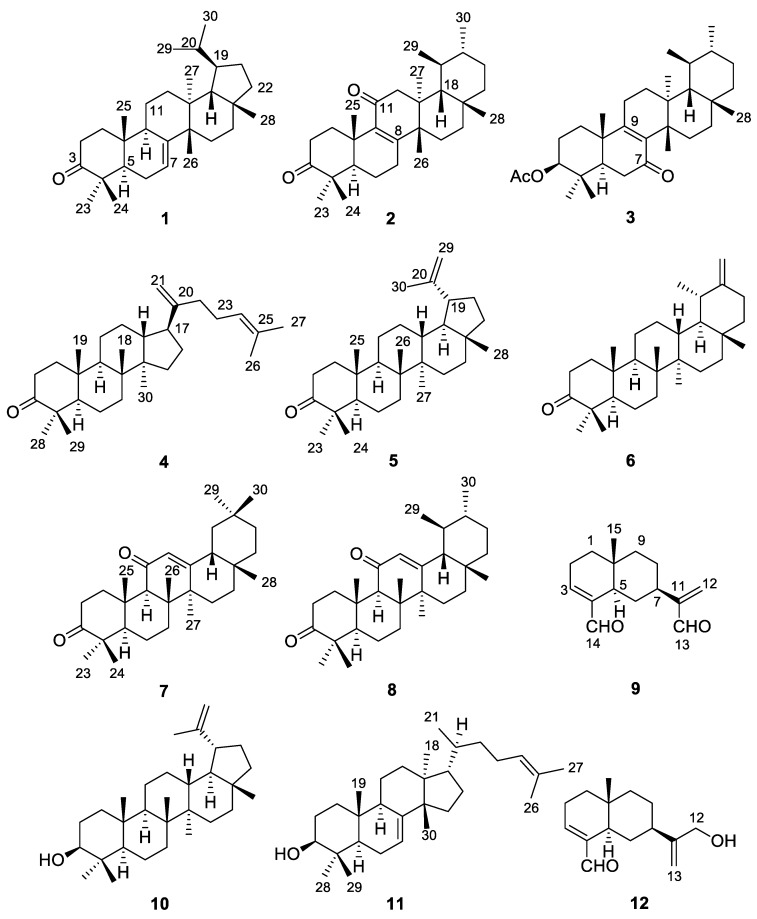
Chemical structures of compounds **1**–**12** from *L. acanthoclada*.

The present study was focused to elucidate the constituents of the Et_2_O soluble portion from the hydroalcoholic extract of the plant. The chemical analysis of this extract revealed the presence of oxygenated terpenoid constituents, which mainly included triterpenoids and sesquiterpenoids, along with fatty acid lipids and sterols. In particular, in this study, three new pentacyclic triterpenoids, named acantholupenone (**1**), acanthobauerendione (**2**) and acanthobauerenone (**3**), as well as nine known sesqui- and triterpenoids **4**–**12** were obtained (Figure 1). The isolation and the chemical characterization of these compounds is described here. 

## 2. Results and Discussion

Roots and aerial parts of *L. acanthoclada* were carefully separated, allowed to dry, and thus, exhaustively extracted with a hydroalcoholic solution. The extracts of each part were evaporated to give two crude residues, which were subsequently transferred to ICB laboratories for the chemical analysis. The Et_2_O soluble portions of the hydroalcoholic extracts of roots and aerial parts were analyzed by comparative TLC chromatography. The secondary metabolite patterns of the two distinct parts revealed to be almost similar and no substantial difference in the relative distribution of the metabolites was observed. Therefore, a portion of the extract of roots was subjected to a first fractionation on silica gel column (see Section 3). ^1^H-NMR spectroscopic analysis evidenced the presence of terpenoid components in four selected fractions. These fractions were subsequently subjected to further purification steps to give three new compounds **1**–**3** and nine known compounds **4**–**12**.

Fraction I (24.7 mg) was constituted of triterpenoids all containing the 3-oxo functionality. In particular, lupenone (**5**) [13,14] and taraxasterone (**6**) [15,16] were the main components of the fraction whereas minor metabolites included new lupenone-related **1** and dammara-20(21),24-dien-3-one **4** [17,18]. Fraction II (14.3 mg) contained four pentacyclic triterpenoids, new bauerane-type compounds **2** and **3** along with olean-12-ene-3,11-dione (**7**) [19,20] and urs-12-ene-3,11-dione (**8**) [19,21], all of which exhibited an enone functional group. Fraction III constituting about 20% of the extract was a mixture of eudesmane dialdehyde **9** [22] and the triterpenoid alcohols lupeol (**10**) [23,24] and tirucalla-7,24-dien-3β-ol (**11**) [25], that were the main metabolites of *L. acanthoclada*. Fraction IV was a complex mixture of eudesmane sesquiterpenoids including **12** [26].

The structures of compounds **1**–**3** were established by extensive spectroscopic analysis (high resolution mass spectrometry (HRMS), nuclear magnetic resonance spectroscopy (NMR), infrared spectroscopy (IR), and ultraviolet spectroscopy (UV)). In particular, NMR experiments of **1**–**3** were conducted in different solvents to get a better resolution with regards to the high field portion of the spectra (Supplementary Materials). Known compounds **4**–**12** were identified by comparison of MS and NMR spectroscopic data with those reported in the literature. The spectroscopic characterization of compounds **8**, **9** and **11** are only partially reported in the literature; the complete NMR assignments of these known molecules have also been achieved in this study (see Section 3).

**Figure 2:**
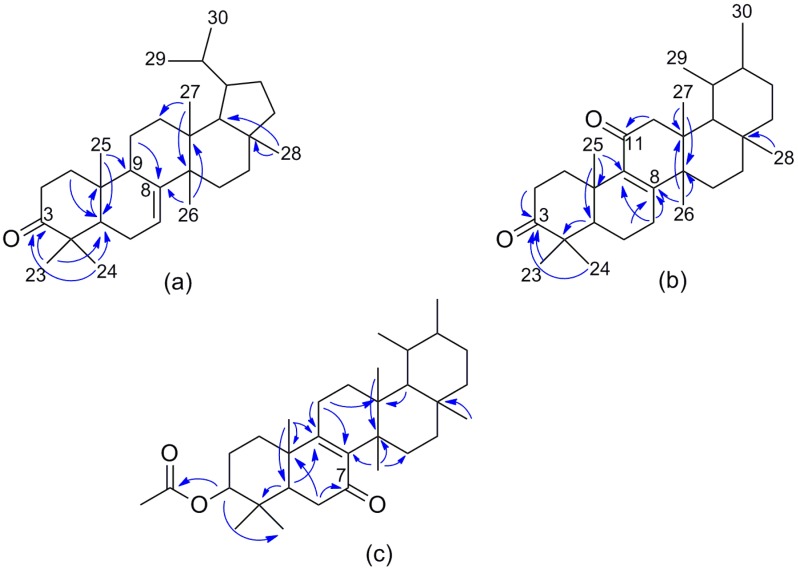
Selected HMBC (blue arrows) for compounds **1** (**a**); **2** (**b**); and **3** (**c**).

Acantholupenone (**1**) was obtained as a white powder. The molecular formula C_30_H_48_O was deduced by the sodium adduct ion at *m*/*z* 447.3598 (M + Na)^+^ in the high resolution electron spray ionization mass spectrum (HRESIMS) accounting for seven indices of hydrogen deficiency. The IR spectrum exhibited typical absorption bands at 1712 and 3039 cm^−1^ suggesting the presence of ketone and double bond functionalities in the structure. Consistent with this, the ^13^C-NMR spectrum (in CDCl_3_) displayed signals due to a carbonyl group (δ_C_ 216.9) and a trisubstituted double bond (δ_C_ 145.4 and δ_C_ 117.2) and all the other resonances between δ_C_ 12.7 and δ_C_ 56.4 assigned to sp^3^ alkyl carbons. This implied that the remaining unsaturation degrees required by the molecular formula should be attributed to five rings. The ^1^H-NMR spectrum (in CDCl_3_) contained six singlet methyls at δ_H_ 0.91 (H_3_-27), 0.92 (H_3_-28), 0.99 (H_3_-25), 1.00 (H_3_-26), 1.04 (H_3_-23), and 1.12 (H_3_-24), and two doublet methyls of an isopropyl group at δ_H_ 0.88 and 0.91 (H_3_-29 and H_3_-30) according to the presence of a pentacyclic 6-6-6-6-5 architecture [27]. Analysis of ^1^H-^1^H correlation spectroscopy (COSY), total correlation spectroscopy (TOCSY) and hetero-nuclear single quantum coherence (HSQC) experiments recorded in both CDCl_3_ (Table 1) and C_5_D_5_N (Section 3) led to the identification of five isolated spin systems: two CH_2_-CH_2_ moieties (rings A and D), a CH-CH_2_-CH (ring B) and a CH-CH_2_-CH_2_ (ring C) fragments, and finally, a sequence constituted by the CH-(CH_3_)_2_ group linked to a CH in turn connected to both a CH and to a CH_2_–CH_2_ unit (ring E). These data strongly suggested that compound **1** had to be either a rearranged lupene derivative, such as tylolupenone [28], or a hancolupenone-like triterpene [29,30,31] with an angular methyl group at C-13 rather than at the C-8 position [27]. The keto function was easily located at C-3 whereas the double bond was positioned at C-7. Careful analysis of hetero-nuclear multiple bond correlation (HMBC) experiments supported the structural assumption. In fact, diagnostic long-range correlations (Figure 2a) were observed from geminal methyls at C-4, H_3_-23 (δ_H_ 1.04) and H_3_-24 (δ_H_ 1.12), to C-3 (δ_C_ 216.9) and C-5 (δ_C_ 51.9), from H-1a (δ_H_ 1.99) to C-5, from H_3_-25 (δ_H_ 0.99) to C-5 and C-9 (δ_C_ 47.9) as well as between H-11a (δ_H_ 1.62) and C-8 (δ_C_ 145.4), H_3_-26 (δ_H_ 1.00) and C-8 and C-13 (δ_C_ 37.7) and, finally, H_3_-27 (δ_H_ 0.91) and C-12 (δ_C_ 32.4) and C-14 (δ_C_ 40.4), consistent with the proposed A-C ring pattern. In addition, in the HMBC spectrum, H_3_-28 (δ_H_ 0.92) showed significant cross-peaks with C-17 (δ_C_ 40.6) and C-18 (δ_C_ 56.4) supporting the D-E ring arrangement.

**Figure 3:**
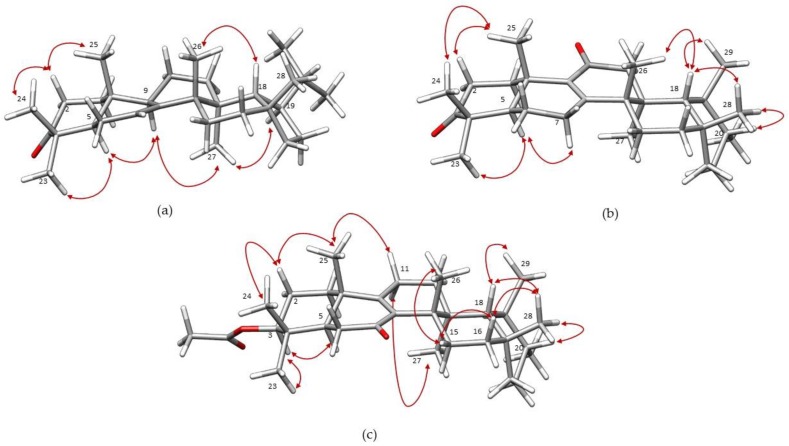
Selected NOE effects (red arrows) for compounds **1** (**a**); **2** (**b**); and **3** (**c**).

**Figure 4:**
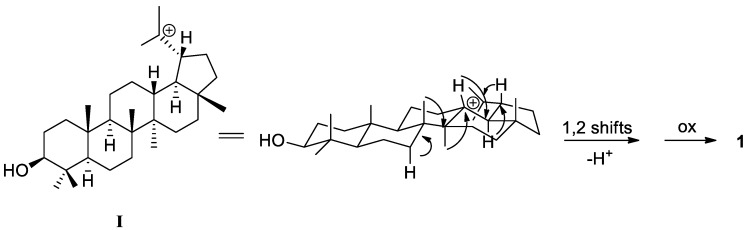
Possible formation of **1** from lupyl cation (**I**).

Once the gross structure of acantholupenone (**1**) was established, the stereochemical aspects were investigated by an extensive analysis of nuclear Overhauser effect spectroscopy (NOESY) and NOE difference experiments recorded in C_5_D_5_N, which provided better resolved ^1^H-NMR spectra. A series of NOE correlations (Figure 3a) were observed between H-2β (δ_H_ 2.74) and both H_3_-24 (δ_H_ 1.05) and H_3_-25 (δ_H_ 0.93) methyl groups as well as between H-5 (δ_H_ 1.71) and H_3_-23 (δ_H_ 1.10) and H-9 (δ_H_ 2.26) inferring the relative configuration of the stereogenic centers at A and B rings. In addition, in the NOESY spectrum, H_3_-27 (δ_H_ 0.98) showed diagnostic cross-peaks with both H-9 and H-19 (δ_H_ 1.62) implying their α-orientation whereas H-18 (δ_H_ 1.53) showed significant correlations with angular H_3_-26 (δ_H_ 1.02) and H_3_-28 (δ_H_ 0.93) methyl groups indicating their β-orientation (Figure 3a). The relative configuration of acantholupenone was thus determined as depicted in structure **1** with a *trans*-C,D and a *cis*-D,E ring junctions, and the α-oriented E-ring. This structural architecture characterizes a small group of rearranged lupene triterpenes, including tylolupenols [28], that have been suggested to be formed from lupyl cation (**I**) by 1,2 shifts following the deprotonation at different positions [27]. According to this, compound **1** should derive by deprotonation at C-7 and sequential migration of H_3_-26, H_3_-27, H-13, H-18 and H-19, as depicted in Figure 4. Acantholupenone is closely related to tylolupenone [28], a synthetic derivative obtained by oxidation of tylolupenols, and differed from this compound in the position of the double bond (∆^7^ rather than ∆^9(11)^). Comparison of NMR data of **1** with tylolupenone and a series of literature model compounds (i.e., pichierenone [32] and swertanone [33]) exhibiting the same A-D ring framework and either ∆^7^ or ∆^9(11)^ double bond, strongly supported our assignment as reported in Table 1.

**Figure 5:**
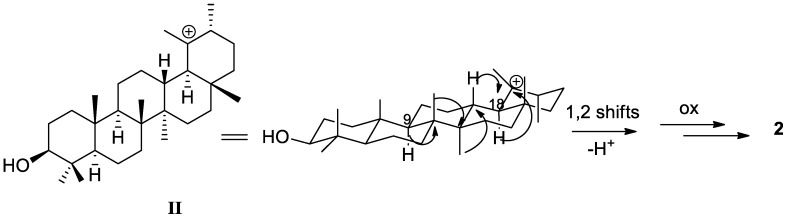
Possible formation of **2** from isoursyl cation (**II**).

Acanthobauerendione (**2**) was obtained as white powder and has the molecular formula C_30_H_46_O_2_ as it was established by the sodium adduct ion at *m*/*z* 461.3392 (M + Na)^+^ in the HRESIMS spectrum. The presence of two ketone groups, one of which α,β-unsaturated, was revealed by IR and UV spectra with typical bands at ν_max_ 1712 and 1657 cm^−1^ and at λ_max_ 255 (log ε 3.56), respectively. According to this, resonances due to a ketone and an enone moiety containing a tetrasubstituted double bond were observed at δ_C_ 217.5 (C, C-3) and δ_C_ 198.0 (C, C-11), 139.5 (C, C-9), and 164.1 (C, C-8) in the carbon spectrum (CDCl_3_, Table 1). The ^1^H- and ^13^C-NMR data of **2** indicated six tertiary methyls [δ_H_ 1.01, δ_C_ 18.1 (H_3_-27); δ_H_ 1.08, δ_C_ 21.8 (H_3_-24); δ_H_ 1.09, δ_C_ 38.3 (H_3_-28); δ_H_ 1.11, δ_C_ 27.6 (H_3_-23); δ_H_ 1.16, δ_C_ 22.0 (H_3_-26); δ_H_ 1.27, δ_C_ 19.8 (H_3_-25)] and two secondary methyls [δ_H_ 0.90, δ_C_ 23.1 (H_3_-30); δ_H_ 1.03, δ_C_ 25.7 (H_3_-29)] (Table 1), suggesting a pentacyclic triterpenoid structure with an ursane-type or rearranged ursane skeleton [27]. In particular, the presence of a bauerane framework [34,35,36], in which the methyl group at C-14 of ursane skeleton is migrated to C-13 and the methyl group at C-8 is migrated to C-14 by 1,2 shifts from isoursyl cation (**II**) (Figure 5) [27], was strongly suspected due to the characteristic carbon value of H_3_-28 appearing abnormally deshielded [37] in triterpenes with this skeleton (i.e., [38,39,40]). 

The inspection of the COSY experiment of **2** aided us to define four proton sequences: two CH_2_-CH_2_, a CH-CH_2_-CH_2_, and a CH-CH(Me)-CH(Me)-CH_2_-CH_2_ spin systems. The presence of an isolated methylene located in α-position to a carbonyl function was detected by NMR signals at δ_H_ 2.26 (s, 2H, H_2_-12) and δ_C_ 49.5 (CH_2_, C-12) (Table 1). A comprehensive analysis of 2D-NMR experiments including COSY, TOCSY, HSQC and HMBC, recorded in both CDCl_3_ and C_5_D_5_N, and the comparison of spectroscopic data with those of related literature compounds (i.e., [38,39,40]) allowed the determination of the planar structure of acanthobauerendione as depicted in formula **2**. Particularly indicative were the HMBC correlations (Figure 2b) that implied the location of the oxo- and enone functionalities at C-3 and C-11, respectively, as well as the obvious position of tetrasubstituted double bond at C-8/C-9. In fact, in the HMBC spectrum (in CDCl_3_), both geminal methyls at C-4, H_3_-23 (δ_H_ 1.11) and H_3_-24 (δ_H_ 1.08), and H-2a (δ_H_ 2.55) showed cross-peaks with C-3 (δ_C_ 217.5), whereas H_2_-12 (δ_H_ 2.26) had correlations with C-11 (δ_C_ 198.0). Finally, correlations were observed from H_2_-7 (δ_H_ 2.45 and 2.14) to C-8 (δ_C_ 164.1) and C-9 (δ_C_ 139.5), from H_3_-25 (δ_H_ 1.27) to C-9 and from both H-6a (δ_H_ 1.72) and H_3_-26 (δ_H_ 1.16) to C-8. The relative configuration of compound **2** was that expected for a bauerane derivative as it was confirmed by a detailed analysis of NOESY and NOE difference experiments, recorded in C_5_D_5_N (significant effects are reported in Figure 3b). Diagnostic NOE effects were observed between H_3_-25 (δ_H_ 1.40) and both H-2β (δ_H_ 2.61) and H_3_-24 (δ_H_ 1.11) as well as between H-5 (δ_H_ 1.68) and both H_3_-23 (δ_H_ 1.17) and H-7α (δ_H_ 2.01) suggesting the relative configuration in rings A and B. Moreover, H-18 (δ_H_ 1.36) showed cross-peaks with H_3_-26 (δ_H_ 1.09), H_3_-28 (δ_H_ 1.03), and H_3_-29 (δ_H_ 0.96) implying all these substituents to be on the same side. . Finally, the NOE correlation between H-20 (δ_H_ 1.48) and H_3_-28 confirmed the expected α-configuration of H_3_-30 according to the bauerane skeleton.

A preliminary analysis of spectroscopic data of acanthobauerenone (**3**) revealed a close structural relationship with compound **2**. The HRESIMS spectrum displayed a sodium adduct ion at *m*/*z* 505.3649 (M + Na)^+^ indicating the molecular formula C_32_H_50_O_3_ with an additional C_2_H_4_O unit with respect to compound **2**. The IR spectrum showed bands at ν_max_ 1656 and 1734 cm^−1^ consistent with the presence of an α,β-unsaturated ketone and an ester carbonyl, respectively. The UV band at λ_max_ 252 (log ε 3.42) supported the presence of the enone moiety, similar to compound **2**. The ^1^H- and ^13^C-NMR spectra (in CDCl_3_) of **3** almost resembled those of **2** exhibiting signals at δ_H_ 0.84 (s), 0.87 (s), 0.91 (d), 0.95 (s), 0.99 (brs), 1.01 (s), 1.06 (s) and 1.22 (s), and at δ_C_ 38.1 (CH_3_), 29.6 (CH_3_), 27.1 (CH_3_), 22.5 (CH_3_), 21.7 (CH_3_), 18.5 (CH_3_), 16.0 (CH_3_), and 15.4 (CH_3_) (Table 1), that were attributed to six tertiary and two secondary methyls in agreement with the bauerane architecture [34,35,36,37,38,39,40]. The 3-oxo functionality in the structure of **2** was replaced in **3** by an acetoxy moiety as revealed by the additional methyl singlet at δ_H_ 2.07 and the 1H double doublet at δ_H_ 4.52 in the proton spectrum, and by signals at δ_C_ 170.9 (C, Ac-*CO*), 79.8 (CH, C-3) and 21.3 (CH_3_, Ac-*CH*_3_) in the carbon spectrum (Table 1). The acetoxy substituent was α-oriented by analysis of the coupling constant values of axial H-3 (dd, *J* = 11.6, 4.1 Hz). The double bond of the enone moiety was tetrasubstituted [δ_C_ 139.3 (C, C-8) and 164.4 (C, C-9)] and necessarily located at C-8/C-9, the same as compound **2**, whereas the α,β-unsaturated carbonyl (δ_C_ 198.4) was located at C-7 by analysis of COSY and TOCSY experiments. The spin systems deduced for rings A–C, which include a CH-CH_2_-CH_2_, a CH-CH_2_, and a CH_2_-CH_2_ fragments, respectively, differed from those of **2** according to a different substitution pattern. Comparison of NMR data of **3** (Table 1) with literature bauerane compounds [34,35,36,37,38,39,40] strongly supported the proposed structure, which was strictly related to isobauerenyl acetate [39,40,41]. Detailed analysis of 2D-NMR experiments, which were recorded also for this compound in both CDCl_3_ and C_5_D_5_N, led us to fully assign proton and carbon resonances (Table 1 and Materials and Methods). In particular, inspection of HMBC spectrum (relevant correlations in Figure 2c) secured the position of the acetoxy substituent and the enone function. Diagnostic correlations were observed from H-3 (δ_H_ 4.52) to Ac-*CO* (δ_C_ 170.9) and C-24 (δ_C_ 16.0), from H-5 (δ_H_ 1.72) to C-4 (δ_C_ 37.6) and C-9 (δ_C_ 164.4), from H_3_-25 (δ_H_ 1.01) to C-5 (δ_C_ 47.4), C-9 and C-10 (δ_C_ 39.2), from H_2_-6 (δ_H_ 2.41 and 2.36) to C-7 (δ_C_ 198.4) and C-10, from H_2_-11 (δ_H_ 2.29 and 2.14) to C-8 (δ_C_ 139.3) and C-9, and finally from H_3_-26 (δ_H_ 1.22) to C-8, C-14 (δ_C_ 40.4), and C-15 (δ_C_ 23.8). A detailed analysis of NOESY and NOE difference experiments, recorded in C_5_D_5_N (significant effects are reported in Figure 3c), confirmed the expected stereochemistry of acanthobauerenone as reported in structure **3**.

In conclusion, we report here the first chemical investigation on the triterpenoid fraction of *L. acanthoclada* providing new insights into the chemistry of plants belonging to the genus *Launaea*. The study led to the characterization of three new triterpenoids **1**–**3**, which were isolated along with known compounds **4**–**12** including triterpenoids with lupane, oleane, ursane, or taraxane skeletons. This finding was in agreement with the literature triterpenoid pattern of other *Launaea* species that have been reported to contain compounds with these structural architectures [7]. It is noteworthy, however, that we also report additional finding in *L. acanthoclada* of irregular frameworks as rearranged lupane (compound **1**) and rearranged ursane (or bauerane) (compounds **2** and **3**) skeletons, that have been never described from *Launaea* species. 

## 3. Materials and Methods

### 3.1. General Experimental Procedures

Optical rotations were obtained with a Jasco P2000 digital polarimeter (JASCO, Tokyo, Japan). UV spectra were acquired on a Jasco V-650 spectrophotometer. IR were recorded on a Jasco FTIR 4100 (JASCO, Tokyo, Japan). NMR experiments were recorded at the NMR Service Centre of the Institute of Biomolecular Chemistry (ICB, CNR). Chemical shifts values are reported in ppm and referenced to the internal signals of residual protons (CDCl_3_, δ_H_ 7.26, δ_C_ 77.0; C_5_D_5_N, δ_H_ 7.19, 7.55, 8.71; δ_C_ 123.5, 135.5, 149.9). 1D- and 2D-NMR spectra were acquired on a Bruker Avance-400 (Bruker Corporation, Billerica, MA, USA) operating at 400 MHz using an inverse probe fitted with a gradient along the *Z*-axis and a Bruker DRX-600 operating (Bruker Corporation, Billerica, MA, USA) at 600 MHz using an inverse TCI CryoProbe fitted with a gradient along the *Z*-axis. ESIMS spectra were measured in positive mode on a Micromass Q-TOF Micro spectrometer (Waters Corporation, Milford, MA, USA) coupled with an HPLC Waters Alliance 2695. HRESIMS spectra were recorded on a Thermo Q-Exactive spectrometer (Thermo Fisher Scientific, Waltham, MA, USA) coupled with a UHPLC Agilent Infinity 1290 (Agilent Technologies, Santa Clara, CA, USA) and on a Shimadzu IT-TOF spectrometer (Shimadzu, Kyoto, Japan) equipped with an ESI interface. HPLC separation was performed on a Shimadzu high-performance liquid chromatography system using a Shimadzu liquid chromatograph (Shimadzu, Kyoto, Japan) LC-10AD equipped with an UV SPD-10A Shimadzu wavelength detector with a reversed-phase (RP) Aventis-Supelco, (Supelco, Bellefonte, PA, USA) column (10 mm × 250 mm). Silica gel chromatography was performed using precoated KieselGel 60 F254 plates (TLC) and Kieselgel 60 powder (70–230 mesh) from Merck (Darmstadt, Germany). The spots on TLC were visualized under UV light (254 nm) and then sprayed with 10% H_2_SO_4_ in water followed by heating.

### 3.2. Plant Material

The plant *L. acanthoclada* was collected in Tilatou, South-East Algeria, during May 2016, and identified by Prof. Bachir Oudjehih, Institute of Agronomy of University of Batna 1 (Algeria). A voucher specimen is deposited in the herbarium of the department of the same University under the number code 123/ISVSA/DA/UHLB1/2016.

### 3.3. Extraction and Isolation

Dried roots (1 kg) and aerial part (400 g) of *L. acanthoclada* were separately macerated with EtOH/H_2_O 7:3 (10 L × 3 and 4 L × 3, respectively). After filtration, the organic solvent was evaporated in vacuo to give two crude residues (77 g for roots and 30 g for aerial parts), which were suspended in H_2_O and partitioned with Et_2_O (500 mL × 3 for roots, 200 mL × 3 for aerial part). The organic phases from roots and aerial parts were evaporated to give the corresponding extracts (11.8 g and 9.0 g, respectively). A portion (2.1 g) of the Et_2_O extract from roots was fractionated by silica-gel column chromatography (column diameter: 5 cm diameter, 120 cm height, 100 g silica-gel) by eluting first with a gradient of Et_2_O in petroleum ether, and subsequently with a gradient of MeOH in CHCl_3_ to obtain eighteen fractions. Four selected fractions were taken into consideration after NMR inspection. Fraction I (24.7 mg), eluted with petroleum ether/Et_2_O 7:3, was subjected to silica-gel column chromatography using a gradient of Et_2_O in petroleum ether to give 11 fractions [I(1)–I(11)]. Subfraction I(5) (8.1 mg) was further purified by reverse-phase HPLC (Phenomenex, Torrance, CA, USA, Kromasil, 5μ, C_18_, 1.0 × 25 cm) with a 20 min gradient from 90% to 100% of MeOH in H_2_O, followed by a 30 min of 100% MeOH (flow rate 1.0 mL/min), to yield pure compounds **4** (0.2 mg, *R*_t_ 31.5 min), **5** (0.8 mg, *R*_t_ 34.2 min), **6** (1.5 mg, *R*_t_ 38.2 min) and **1** (0.3 mg, *R*_t_ 41.1 min). Fraction II (22.2 mg), eluted with petroleum ether/Et_2_O 6:4, was fractionated on C18 cartridge (SPE, Macherey-Nagel, Düren, Germany) eluted with a gradient of MeOH in H_2_O to give 3 subfractions [II(1)–II(3)]. Subfractions II(2) (8.6 mg) was further purified by reverse-phase HPLC (Phenomenex, Kromasil, 5μ, C_18_, 1.0 × 25 cm) with a 50 min gradient from 90% to 100% of MeOH in H_2_O to yield pure compounds **2** (1.4 mg, *R*_t_ 21.4 min), **7** (1.2 mg, *R*_t_ 22.3 min), **8** (1.3 mg, *R*_t_ 22.9 min) and **3** (1.5 mg, *R*_t_ 30.1 min). An aliquot (20.0 mg) of fraction III (400 mg) (eluted with petroleum ether/Et_2_O, 1:1 from the first column) was purified on a C18 cartridge (SPE, Macherey-Nagel) by using a gradient of MeOH in H_2_O to get 4 subfractions [III(1)–III(4)]. Subfraction III(2), eluted with MeOH/H_2_O, 7:3, contained pure compound **9** (1.1 mg), whereas subfraction III(4) (13.0 mg), eluted with MeOH, was further purified by reverse-phase HPLC (Phenomenex, Kromasil, 5μ, C_18_, 1.0 × 25 cm) using MeOH in isocratic mode to give pure compounds **10** (1.2 mg, *R*_t_ 22.6 min) and **11** (1.0 mg, *R*_t_ 24.8 min). Fraction IV (58.9 mg), eluted with petroleum ether/Et_2_O, 3:7, was additionally fractionated on a C18 cartridge (SPE, Macherey-Nagel) with a gradient of MeOH in H_2_O to give pure compound **12** (1.0 mg), eluted with MeOH/H_2_O 4:6.

*Acantholupenone* (**1**). White powder; [α]${}_{D}^{25}$ −48.5 (c 0.02, CHCl_3_); UV (MeOH) λ_max_ (log ε) 276 (2.83); IR (KBr) ν_max_ 3039, 2855, 1712, 1458, 1378, 810, 723 cm**^−^**^1^; ^1^H- and ^13^C-NMR (CDCl_3_) see Table 1; ^1^H-NMR (C_5_D_5_N, 600 MHz) δ 5.55 (1H, brd, *J* = 2.7 Hz, H-7), 2.74 (1H, ddd, *J* = 14.6, 14.6, 5.6 Hz, H-2β), 2.27 (1H, m, H-2α), 2.26 (1H, m, H-9α), 2.01 (2H, m, H_2_-6), 1.84 (1H, ddd, *J* = 12.6, 4.3, 3.6 Hz, H-1β), 1.76 (2H, m, H_2_-12), 1.74 (1H, m, H-22a), 1.71 (1H, m, H-5α), 1.62 (1H, m, H-19α), 1.56 (2H, m, H_2_-15), 1.54 (2H, m, H_2_-16), 1.53 (2H, m, H-20 and H-18β), 1.50 (2H, m, H_2_-11), 1.47 (2H, m, H_2_-21), 1.33 (1H, m, H-1α), 1.14 (1H, m, H-22b), 1.10 (3H, s, H_3_-23), 1.05 (3H, s, H_3_-24), 1.02 (3H, s, H_3_-26), 0.98 (3H, s, H_3_-27), 0.96 (3H, d, *J* = 6.2 Hz, H_3_-30), 0.93 (6H, s, H_3_-25 and H_3_-28), 0.92 (3H, d, *J* = 6.2 Hz, H_3_-29); ^13^C-NMR (C_5_D_5_N, 150 MHz) δ 215.7 (CO, C-3), 145.6 (C, C-8), 117.8 (CH, C-7), 57.1 (CH, C-18), 51.8 (CH, C-5), 50.3 (CH, C-19 ), 48.2 (CH, C-9), 48.0 (C, C-4), 41.5 (C, C-14), 40.0 (C, C-17), 39.3 (CH_2_, C-22), 38.4 (CH_2_, C-1), 37.2 (C, C-13), 36.5 (CH, C-20), 35.4 (CH_2_, C-2), 34.6 (CH_2_, C-16), 34.2 (C, C-10), 33.6 (CH_3_, C-28), 30.0 (CH_2_, C-12), 29.3 (CH_2_, C-21), 29.1 (CH_2_, C-15), 24.7 (CH_2_, C-6), 24.5 (CH_3_, C-23), 23.8 (CH_3_, C-30), 23.3 (CH_3_, C-27), 23.2 (CH_3_, C-26), 22.3 (CH_3_, C-29), 21.7 (CH_3_, C-24), 17.1 (CH_2_, C-11), 13.1 (CH_3_, C-25); ESI MS *m*/*z* 447 [M + Na]^+^; HR ESIMS *m*/*z* 447.3598 [M + Na]^+^ (calcd. for C_30_H_48_ONa 447.3603).

*Acanthobauerendione* (**2**)*.* White powder; [α]${}_{D}^{25}$ –4.8 (c 0.04, CHCl_3_); UV (MeOH) λ_max_ (log ε) 255 (3.56); IR (KBr) ν_max_ 2950, 1712, 1657, 1461, 1378, 1263, 967, 805 cm**^−^**^1^; ^1^H- and ^13^C-NMR (CDCl_3_) see Table 1; ^1^H-NMR (C_5_D_5_N, 600 MHz) δ 3.01 (1H, dt, *J* = 13.0, 6.8 Hz, H-1β), 2.61 (1H, m, H-2β), 2.39 (1H, m, H-2α), 2.35 (2H, ABq, *J* = 18.7 Hz , H_2_-12), 2.33 (1H, m, H-7β), 2.01 (1H, ddd, *J* = 12.3, 11.7, 7.5 Hz, H-7α), 1.68 (1H, dd, *J* = 13.0, 2.0 Hz, H-5α), 1.62 (1H, m, H-15a), 1.60 (2H, m, H-6α and H-1α), 1.56 (1H, m, H-22a), 1.48 (1H, m, H-20β ),1.46 (1H, m, H-16β ), 1.40 (3H, s, H_3_-25), 1.36 (1H, m, H-18β), 1.34 (1H, m, H-6β), 1.28 (1H, m, H-15b), 1.20 (1H, m, H-22b), 1.17 (3H, s, H_3_-23), 1.15 (1H, m, H-16α), 1.11 (3H, s, H_3_-24), 1.10 (2H, m, H_2_-21), 1.09 (3H, s, H_3_-26), 1.03 (3H, s, H_3_-28), 0.96 (3H, d, overlap, H_3_-29), 0.96 (1H, m, H-19α), 0.95 (3H, s, H_3_-27), 0.89 (3H, d, *J* = 5.8 Hz, H_3_-30); ^13^C-NMR (C_5_D_5_N, 150 MHz) δ 217.0 (CO, C-3), 197.8 (CO, C-11), 164.4 (C, C-8), 139.4 (C, C-9), 52.3 (CH, C-18), 51.3 (CH, C-5 ), 49.6 (CH_2_, C-12), 47.0 (C, C-4), 43.4 (C, C-14), 40.7 (C, C-13), 37.4 (CH_2_, C-16), 37.3 (CH_3_, C-28), 36.5 (C, C-10), 35.1 (CH_2_, C-1), 35.0(CH, C-19), 34.3 (CH_2_, C-2), 32.2 (CH_2_, C-17), 32.0 (CH_2_, C-22), 31.6 (CH, C-20), 29.3 (CH_2_, C-21), 28.4 (CH_2_, C-7), 26.8 (CH_3_, C-23), 25.9 (CH_2_, C-15), 24.8 (CH_3_, C-29), 22.5 (CH_3_, C-30), 21.0 (2 × CH_3_, C-24 and C-26), 19.5 (CH_3_, C-25), 19.4 (CH_2_, C-6), 18.2 (CH_3_, C-27); ESI MS *m*/*z* 461 [M + Na]^+^; HR ESIMS *m*/*z* 461.3392 [M + Na]^+^ (calcd. for C_30_H_46_O_2_Na 461.3396).

*Acanthobauerenone* (**3**). White powder; [α]${}_{D}^{25}$ +4.1 (c 0.13, CHCl_3_); UV (MeOH) λ_max_ (log ε) 252 (3.42); IR (KBr) ν_max_ 2949, 1734, 1656, 1597, 1459, 1370, 1243, 977 cm**^−^**^1^; ^1^H- and ^13^C-NMR (CDCl_3_) see Table 1; ^1^H-NMR (C_5_D_5_N, 600 MHz) δ 4.72 (1H, dd, *J* = 11.5, 4.2 Hz, H-3α), 2.70 (1H, ddd, *J* = 13.0, 4.3, 2.5 Hz, H-15β), 2.55 (1H, dd, *J* = 18.7, 5.9 Hz, H-6a), 2.49 (1H, dd, *J* = 18.7, 13.0 Hz, H-6b), 2.16 (1H, m, H-11α), 2.06 (1H, m, H-11β), 2.05 (3H, s, CO*CH*_3_), 1.82 (1H, dd, *J* = 13.0, 5.9 Hz, H-5α), 1.80 (2H, m, H-21a and H-2a), 1.71 (1H, m, H-2b), 1.70 (1H, m, H-1β), 1.64 (1H, m, H-22a), 1.60 (1H, ddd, *J* = 14.5, 13.8, 4.3 Hz, H-16β), 1.53 (1H, m, H-20β), 1.49 (1H, ddd, *J* = 14.0, 12.0, 4.3 Hz, H-15α), 1.41 (1H, m, H-21b), 1.39 (1H, m, H-1α), 1.37 (3H, s, H_3_-26), 1.34 (3H, m, H-18β and H_2_-12), 1.19 (1H, m, H-22b), 1.18 (1H, m, H-16α), 1.04 (1H, m, H-19α), 1.04 (3H, s, H_3_-28), 1.00 (3H, d, *J* = 6.4 Hz, H_3_-29), 0.95 (3H, s, H_3_-25), 0.93 (3H, s, H_3_-24), 0.92 (3H, brs, H_3_-30), 0.88 (3H, s, H_3_-27), 0.83 (3H, s, H_3_-23); ^13^C-NMR (C_5_D_5_N, 150 MHz) δ 197.7 (CO, C-7), 170.5 (C, *CO*CH_3_), 164.3 (C, C-9), 139.6 (C, C-8), 79.8 (CH, C-3), 51.6 (CH, C-18 ), 47.5 (CH, C-5), 40.7 (C, C-14), 39.4 (C, C-10), 38.9 (CH_3_, C-28), 38.7 (C, C-13), 37.8 (C, C-4), 36.7 (CH_2_, C-16), 36.2 (CH_2_, C-6), 34.0 (CH, C-19), 33.5 (CH_2_, C-1), 32.1 (CH_2_, C-22), 31.7 (CH, C-20), 31.5 (C, C-17), 28.2 (2 × CH_2_, C-21 and C-12), 27.0 (CH_3_, C-23), 25.3 (CH_3_, C-29), 24.0 (CH_2_, C-2), 23.7 (CH_2_, C-15), 22.5 (CH_2_, C-11), 22.0 (CH_3_, C-30), 21.0 (CH_3_, C-26), 20.7 (CH_3_, CO*CH*_3_), 18.3 (CH_3_, C-25), 16.1 (CH_3_, C-24), 15.7 (CH_3_, C-27); ESI MS *m*/*z* 505 [M + Na]^+^; HR ESIMS *m*/*z* 505.3649 [M + Na]^+^ (calcd. for C_32_H_50_O_3_Na 505.3658).

*Urs-12-ene-3*,*11-dione* (**8**). ^1^H-NMR (CDCl_3_, 400 MHz) δ 5.58 (1H, s, H-12), 2.94 (1H, m, H-1a), 2.62 (1H, m, H-2a), 2.42 (1H, s, H-9), 2.37 (1H, m, H-2b), 2.10 (1H, m, H-15a), 1.90 (1H, ddd, *J* = 18.7, 13.5, 5.4 Hz, H-15b), 1.88 (1H, m, H-16a), 1.70 (1H, m, H-7a), 1.55 (1H, m, H-18), 1.54 (2H, m, H_2_-6), 1.49 (1H, m, H-22a ), 1.47 (1H, m, H-7b ), 1.41 (1H, m, H-1b), 1.39 (1H, m, H-19), 1.34 (1H, m, H-20), 1.31 (1H, m, H-22b), 1.30 (6H, brs, H_3_-25 and H_3_-27), 1.27 (1H, m, H-5), 1.26 (2H, m, H_2_-21), 1.21 (3H, s, H_3_-26), 1.10 (3H, s, H_3_-24), 1.07 (3H, s, H_3_-23), 1.01 (1H, m, H-16b), 0.95 (3H, brs, H_3_-30), 0.83 (3H, s, H_3_-28), 0.80 (3H, d, *J* = 6.5 Hz, H_3_-29); ^13^C-NMR (CDCl_3_, 150 MHz) δ 216.8 (CO, C-3), 198.7 (CO, C-11), 164.9 (C, C-13), 130.1 (C, C-12), 60.5 (CH, C-9), 58.9 (CH, C-18 ), 55.3 (CH, C-5), 47.5 (C, C-4), 44.3 (C, C-8), 43.8 (C, C-14), 40.6 (CH_2_, C-22), 39.5 (CH_2_, C-1), 39.0 (CH, C-19), 38.9 (CH, C-20), 36.4 (C, C-10), 33.7 (C, C-17), 33.9 (CH_2_, C-2), 31.8 (CH_2_, C-7), 31.7 (CH_2_, C-21), 28.6 (CH_3_, C-28), 27.5 (CH_2_, C-16), 27.1 (CH_2_, C-15), 26.0 (CH_3_, C-24), 21.1 (CH_3_, C-23), 20.9 (CH_3_, C-30), 20.3 (CH_3_, C-27 ), 18.1 (CH_2_, C-6), 17.7 (CH_3_, C-26), 17.1 (CH_3_, C-29), 15.4 (CH_3_, C-25); ESI MS *m*/*z* 461 [M + Na]^+^.

*Eudesmane dialdehyde* (**9**). ^1^H-NMR (CDCl_3_, 600 MHz) δ 9.52 (1H, s, H-13), 9.41 (1H, s, H-15), 6.71 (1H, m, H-3), 6.26 (1H, brs, H-12), 5.96 (1H, s, H-12), 2.73 (1H, m, H-6a), 2.60 (1H, m, H-7), 2.40 (2H, m, H_2_-2), 2.30 (1H, m, H-5), 1.68 (1H, m, H-8a), 1.52 (1H, m, H-9a), 1.51 (1H, m, H-8b), 1.48 (1H, m, H-1a), 1.39 (1H, m, H-1b), 1.37 (1H, m, H-9b), 1.24 (1H, m, H-6b), 0.85 (3H, s, H_3_-14); ^13^C-NMR (CDCl_3_, 150 MHz) δ 194.6 (CHO, C-15), 194.3 (CHO, C-13), 154.6 (C, C-11), 153.1 (CH, C-3), 141.8 (C, C-4), 133.1 (CH_2_, C-12 ), 43.5 (CH, C-5), 39.5 (CH_2_, C-9), 37.0 (CH, C-7), 36.4 (CH_2_, C-1), 32.1 (C, C-10), 27.0 (CH_2_, C-8), 26.3 (CH_2_, C-6), 24.4 (CH_2_, C-2), 15.7 (CH_3_, C-15); ESI MS *m*/*z* 255 [M + Na]^+^.

*Tirucalla-7*,*24-dien-3β-ol* (**11**). ^1^H-NMR (CDCl_3_, 600 MHz) δ 5.25 (1H, brs, H-7), 5.10 (1H, m, H-24), 3.24 (1H, dd, *J* = 11.5, 3.9 Hz, H-3), 2.19 (1H, m, H-9), 2.15 (1H, m, H-6a), 2.04 (1H, m, H-23a), 1.97 (1H, m, H-6b), 1.93 (1H, m, H-16a), 1.86 (1H, m, H-23b), 1.80 (1H, m, H-12a), 1.79 (1H, m, H-15a), 1.70 (1H, m, H-2a ), 1.68 (3H, s, H_3_-26 ), 1.67 (1H, m, H-1a), 1.61 (1H, m, H-2b), 1.60 (3H, s, H_3_-27), 1.58 (2H, m, H_2_-22), 1.50 (2H, m, H_2_-11), 1.47 (1H, m, H-17), 1.45 (1H, m, H-15b), 1.43 (1H, s, H-20), 1.30 (2H, m, H-5 and H-16b), 1.25 (1H, m, H-12b), 1.14 (1H, m, H-1b), 0.97 (6H, s, H_3_-30 and H_3_-29), 0.86 (3H, s, H_3_-28), 0.84 (3H, d, *J* = 6.2 Hz, H_3_-21), 0.80 (3H, s, H_3_-18), 0.74 (3H, s, H_3_-19); ^13^C-NMR (CDCl_3_, 150 MHz) δ 145.9 (C, C-8), 130.7 (C, C-25), 124.8 (CH, C-24), 117.9 (CH, C-7), 78.9 (CH, C-3), 52.8 (CH, C-17), 50.3 (CH, C-5), 49.0 (CH, C-9), 48.1 (C, C-15), 43.0 (C, C-13), 38.0 (C, C-4), 37.2 (CH_2_, C-1), 34.4 (C, C-10), 34.1 (CH, C-20), 33.8 (CH_2_, C-22), 33.6 (CH_2_, C-12), 33.5 (CH_2_, C-15), 28.6 (CH_2_, C-16), 27.3 (2 × CH_3_, C-30 and C-29), 25.6 (CH_3_, C-26), 25.3 (CH_2_, C-2), 25.2 (CH_2_, C-23), 23.7 (CH_2_, C-6), 21.0 (CH_3_, C-18), 18.9 (CH_3_, C-21), 18.0 (CH_2_, C-11), 17.4 (CH_3_, C-27), 14.3 (CH_3_, C-28), 13.4 (CH_3_, C-19); ESI MS *m*/*z* 449 [M + Na]^+^.

## Figures and Tables

**Table 1:** ^1^H- and ^13^C-NMR spectral data ^a^ of **1**–**3** in CDCl_3_.

Position	1		2		3	
	^13^C	^1^H (*J*, Hz)	^13^C	^1^H (*J*, Hz)	^13^C	^1^H (*J*, Hz)
1a	38.3, CH_2_	1.99, m	35.2, CH_2_	2.79, ddd (13.5, 7.6, 4.5)	33.9, CH_2_	2.30, m
1b		1.43, m		1.44, m		1.84, m
2a	35.0, CH_2_	2.74, ddd (14.4, 14.4, 5.5)	34.8, CH_2_	2.55, m	25.1, CH_2_	1.71, m
2b		2.24, m		2.48, m		1.54, m
3	216.9, C		217.5, C		79.8, CH	4.52, dd (11.6, 4.1)
4	47.7, C		47.3, C		37.6, C	
5	51.9, CH	1.69, dd (9.9, 7.9)	51.6, CH	1.60, m	47.4, CH	1.72, dd (12.5, 6.8)
6a	24.4, CH_2_	2.11 m	21.6, CH_2_	1.72, m	36.4, CH_2_	2.41, dd (18.6, 6.8)
6b				1.48, m		2.36, dd (18.6, 12.5)
7a	117.2, CH	5.53 dd (6.4, 3.2)	30.5, CH_2_	2.45, m	198.4, C	
7b				2.14, m		
8	145.4, C		164.1, C		139.3, C	
9	47.9, CH	2.24, m	139.5, C		164.4, C	
10	35.4, C		37.1, C		39.2, C	
11a	16.6, CH_2_	1.62, m	198.0, C		23.6, CH_2_	2.29, m
11b		1.54, m				2.14, m
12a	32.4, CH_2_	1.54, m	49.5, CH_2_	2.26, s	29.6, CH_2_	1.50, m
12b						1.40, m
13	37.7, C		39.6, C		38.4, C	
14	40.4, C		43.1, C		40.4, C	
15a	28.8, CH_2_	1.32, m	26.1, CH_2_	1.14, m	23.8, CH_2_	1.38, m
15b				1.60, m		
16a	34.7, CH_2_	1.49, m	36.6, CH_2_	1.57, m	37.8, CH_2_	1.55, m
16b				1.27, m		1.17, m
17	40.6, C		32.3, C		31.5, C	
18	56.4, CH	1.50, m	52.2, CH	1.42, brs	51.5, CH	1.33, brd (2.3)
19	49.7, CH	1.58, m	36.2, CH	1.02, m	36.0, CH	1.03, m
20	35.0, CH	1.55, m	31.0, CH	1.40, m	33.1, CH	1.59, m
21a	28.6, CH_2_	1.77, m	28.6, CH_2_	1.60, m	27.9, CH_2_	1.67, m
21b		1.51, m		1.31,m		
22a	38.6, CH_2_	1.75, m	31.5, CH_2_	1.26, m	31.8, CH_2_	1.55, m
22b		1.17, m				1.26, m
23	24.7, CH_3_	1.04, s	27.6, CH_3_	1.11, s	29.6, CH_3_	0.87, s
24	21.5, CH_3_	1.12, s	21.8, CH_3_	1.08, s	16.0, CH_3_	0.95, s
25	12.7, CH_3_	0.99, s	19.8, CH_3_	1.27, s	18.5, CH_3_	1.01, s
26	23.5, CH_3_	1.00, s	22.0, CH_3_	1.16, s	21.7, CH_3_	1.22, s
27	23.2, CH_3_	0.91, s	18.1, CH_3_	1.01, s	15.4, CH_3_	0.84, s
28	33.1, CH_3_	0.92, s	38.3, CH_3_	1.09, s	38.1, CH_3_	1.06, s
29	22.0, CH_3_	0.88, d (6.3)	25.7, CH_3_	1.03, brs	27.1, CH_3_	0.99, brs
30	23.2, CH_3_	0.91, d (6.0)	23.1, CH_3_	0.90, d (5.9)	22.5, CH_3_	0.91, d (5.9)
Ac-*CO*					170.9, C	
Ac-*CH*_3_					21.3, CH_3_	2.07, s

^a^ Assignments aided by COSY, TOCSY, HSQC, HMBC (*J* = 7 and 10 Hz).

## Supplementary Materials

The following are available online: 1D- and 2D-NMR, and HRESIMS spectra of compounds **1**–**3**.
