# Supplementary material for: Exploring the Chemical Diversity of Algerian Plants: Three New Pentacyclic Triterpenoids from Launaea acanthoclada Roots
Source: Molecules. 2017 Dec 30;23(1):80. doi: 10.3390/molecules23010080 (PMC6017627; doi:10.3390/molecules23010080)
Supplement: Supplementary file 1 [file molecules-23-00080-s001.pdf]

# Exploring the Chemical Diversity of Algerian Plants*:* Three New Pentacyclic Triterpenoids from *Launaea acanthoclada* roots

Nabila Zergainoh[1,2], Maria Letizia Ciavatta[1*], Marianna Carbone[1], Fatma Bitam[3], Mohamed Cherif Aberkane[2] and Margherita Gavagnin[1]

[1] Consiglio Nazionale delle Ricerche (CNR), Istituto di Chimica Biomolecolare (ICB), Via Campi Flegrei, 34, 80078 Pozzuoli (Naples), Italy

[2] Université de Batna 1, Faculté des Sciences de la Matière, Département de Chimie, Laboratoire de Chimie et Chimie de l'Environnement (L.C.C.E), Batna, Algérie

[3] Université de Batna 2, Faculté de Médecine, Département de Pharmacie, Batna, Algérie

* Correspondence: lciavatta@icb.cnr.it (M.L.C)

*S1.* $^{1}$H NMR spectrum of acantholupenone (**1**) (600 MHz, $CDCl_3$).

*S2.* $^{13}$C NMR spectrum of acantholupenone (**1**) (150 MHz, $CDCl_3$).

*S3.* COSY spectrum of acantholupenone (**1**) (600 MHz, $CDCl_3$).

*S4.* HSQC spectrum of acantholupenone (**1**) (600 MHz, $CDCl_3$).

*S5.* HMBC spectrum of acantholupenone (**1**) (600 MHz, $CDCl_3$).

*S6.* TOCSY spectrum of acantholupenone (**1**) (400 MHz, $CDCl_3$).

*S7.* $^{1}$H NMR spectrum of acantholupenone (**1**) (600 MHz, $C_5D_5N$).

*S8.* COSY spectrum of acantholupenone (**1**) (600 MHz, $C_5D_5N$).

*S9.* edHSQC spectrum of acantholupenone (**1**) (600 MHz, $C_5D_5N$).

*S10.* HMBC spectrum of acantholupenone (**1**) (600 MHz, $C_5D_5N$).

*S11.* NOESY spectrum of acantholupenone (**1**) (400 MHz, $C_5D_5N$).

*S12.* HRESIMS spectrum of acantholupenone (**1**).

*S13.* $^{1}$H NMR spectrum of acanthobauerendione (**2**) (600 MHz, $CDCl_3$).

*S14*. $^{13}$C NMR spectrum of acanthobauerendione (**2**) (150 MHz, $CDCl_3$).

*S15*. COSY spectrum of acanthobauerendione (**2**) (600 MHz, $CDCl_3$).

*S16*. edHSQC spectrum of acanthobauerendione (**2**) (400 MHz, $CDCl_3$).

*S17*. HMBC spectrum of acanthobauerendione (**2**) (600 MHz, $CDCl_3$).

*S18*. TOCSY spectrum of acanthobauerendione (**2**) (600 MHz, $CDCl_3$).

*S19*. $^{1}$H NMR spectrum of acanthobauerendione (**2**) (600 MHz, $C_5D_5N$).

*S20*. $^{13}$C NMR spectrum of acanthobauerendione (**2**) (150 MHz, $C_5D_5N$).

*S21*. COSY spectrum of acanthobauerendione (**2**) (600 MHz, $C_5D_5N$).

*S22*. edHSQC spectrum of acanthobauerendione (**2**) (600 MHz, $C_5D_5N$).

*S23*. HMBC spectrum of acanthobauerendione (**2**) (600 MHz, $C_5D_5N$).

*S24*. NOESY spectrum of acanthobauerendione (**2**) (400 MHz, $C_5D_5N$).

*S25*. HRESIMS spectrum of acanthobauerendione (**2**).

*S26*. $^{1}$H NMR spectrum of acanthobauerenone (**3**) (600 MHz, $CDCl_3$).

*S27*. $^{13}$C NMR spectrum of acanthobauerenone (**3**) (150 MHz, $CDCl_3$).

*S28*. COSY spectrum of acanthobauerenone (**3**) (600 MHz, $CDCl_3$).

*S29*. edHSQC spectrum of acanthobauerenone (**3**) (600 MHz, $CDCl_3$).

*S30*. HMBC spectrum of acanthobauerenone (**3**) (600 MHz, $CDCl_3$).

*S31*. $^{1}$H NMR spectrum of acanthobauerenone (**3**) (600 MHz, $C_5D_5N$).

*S32*. $^{13}$C NMR spectrum of acanthobauerenone (**3**) (150 MHz, $C_5D_5N$).

*S33*. COSY spectrum of acanthobauerenone (**3**) (600 MHz, $C_5D_5N$).

*S34*. edHSQC spectrum of acanthobauerenone (**3**) (600 MHz, $C_5D_5N$).

*S35*. HMBC spectrum of acanthobauerenone (**3**) (600 MHz, $C_5D_5N$).

*S36*. TOCSY spectrum of acanthobauerenone (**3**) (400 MHz, $C_5D_5N$).

*S37*. NOESY spectrum of acanthobauerenone (**3**) (400 MHz, $C_5D_5N$).

*S38*. HRESIMS spectrum of acanthobauerenone (**3**).

*S39*. NMR data in $C_5D_5N$ of acantholupenone (**1**) acanthobauerendione (**2**), and acanthobauerenone (**3**).

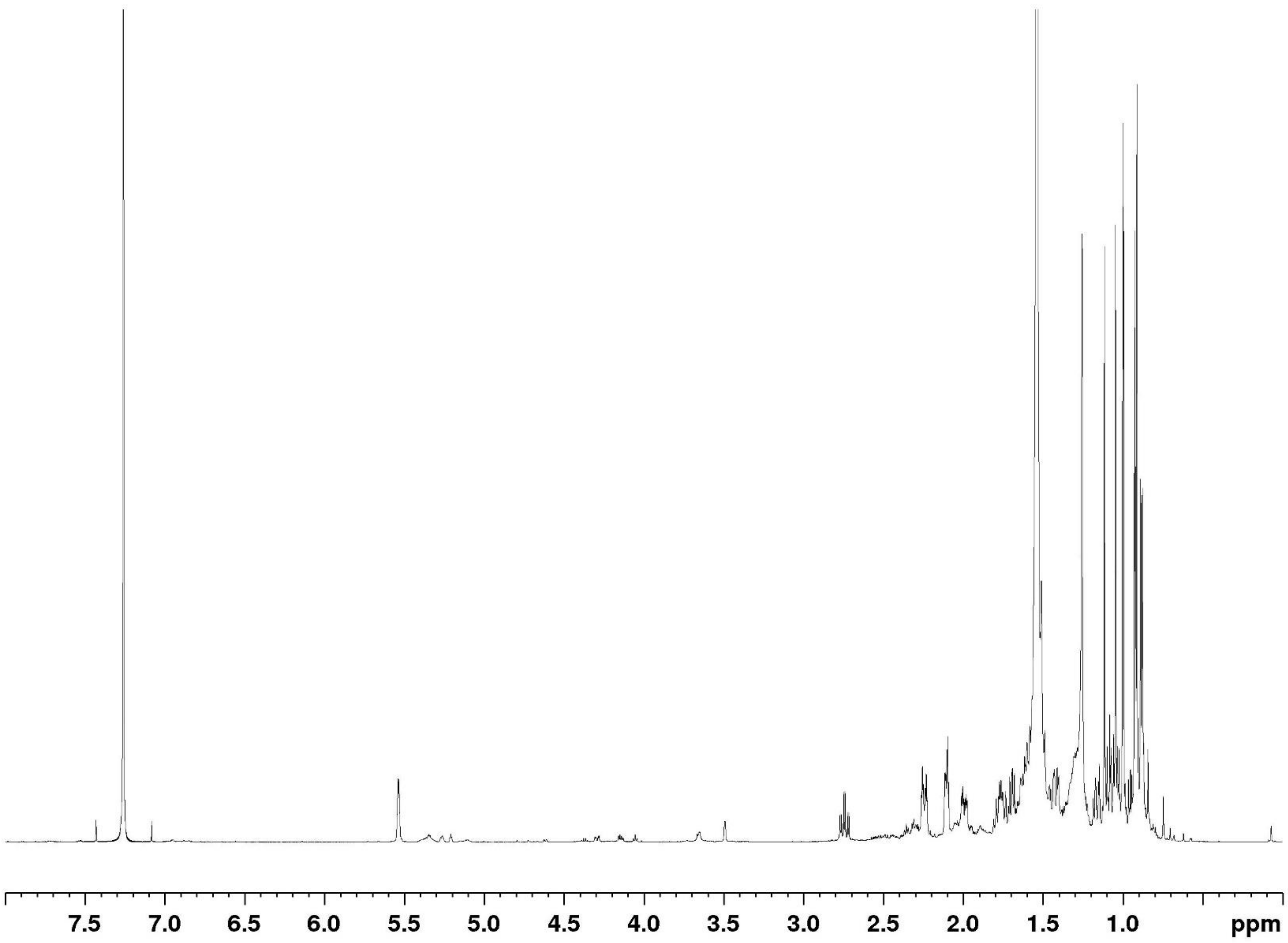


$^1$H NMR spectrum of acantholupenone (**1**) (600 MHz, $CDCl_3$)

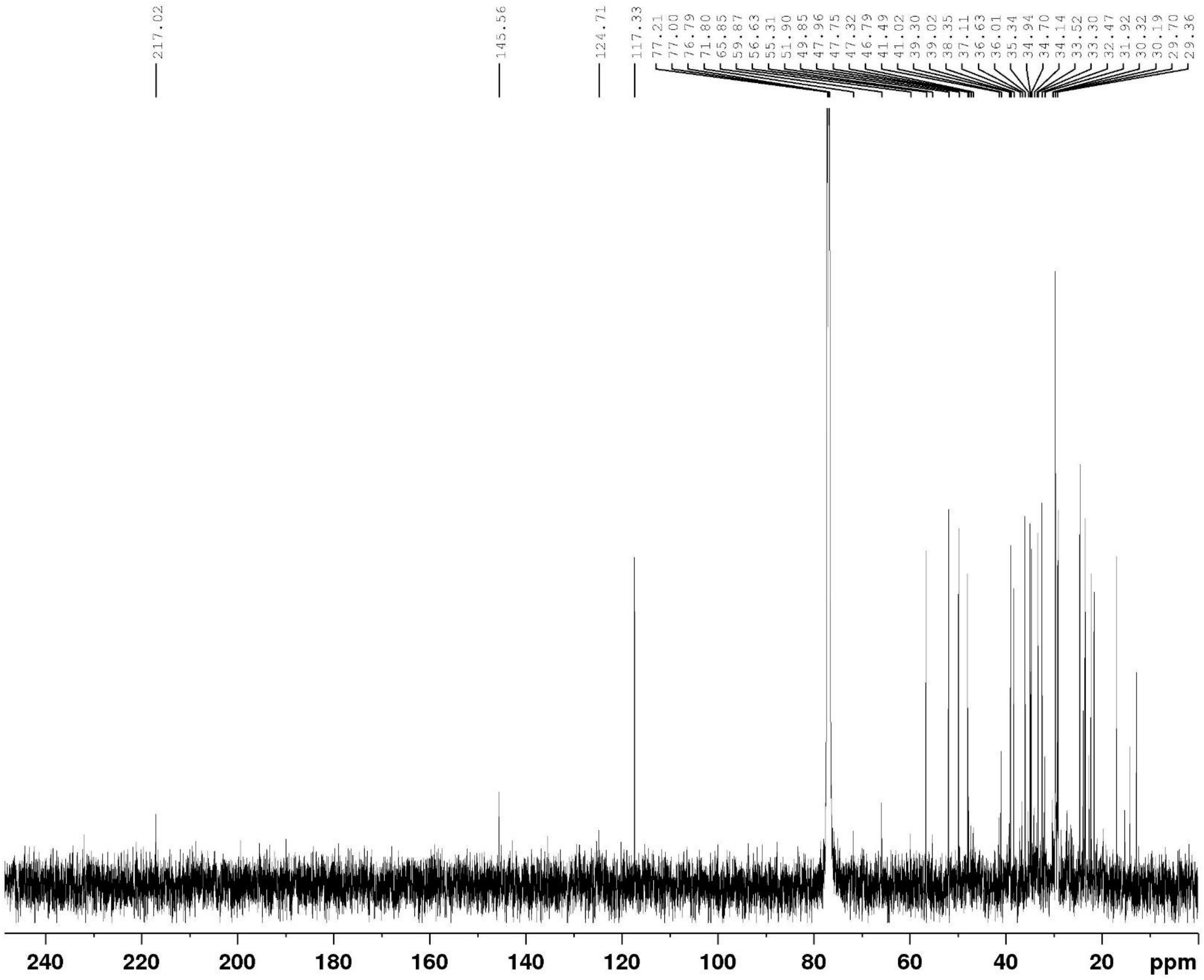


$^{13}$C NMR spectrum of acantholupenone (**1**) (150 MHz, $CDCl_3$).

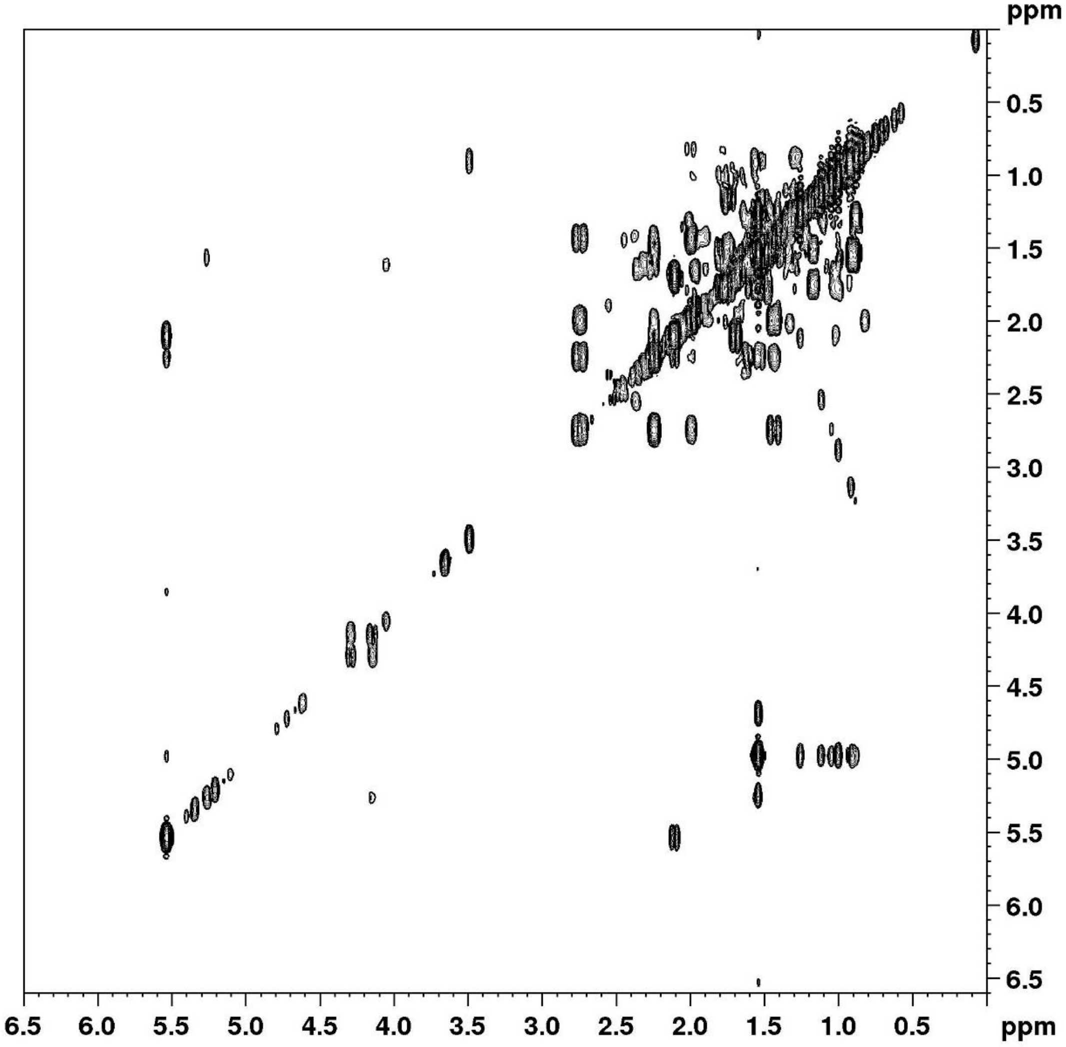


COSY spectrum of acantholupenone (**1**) (600 MHz, $CDCl_3$).

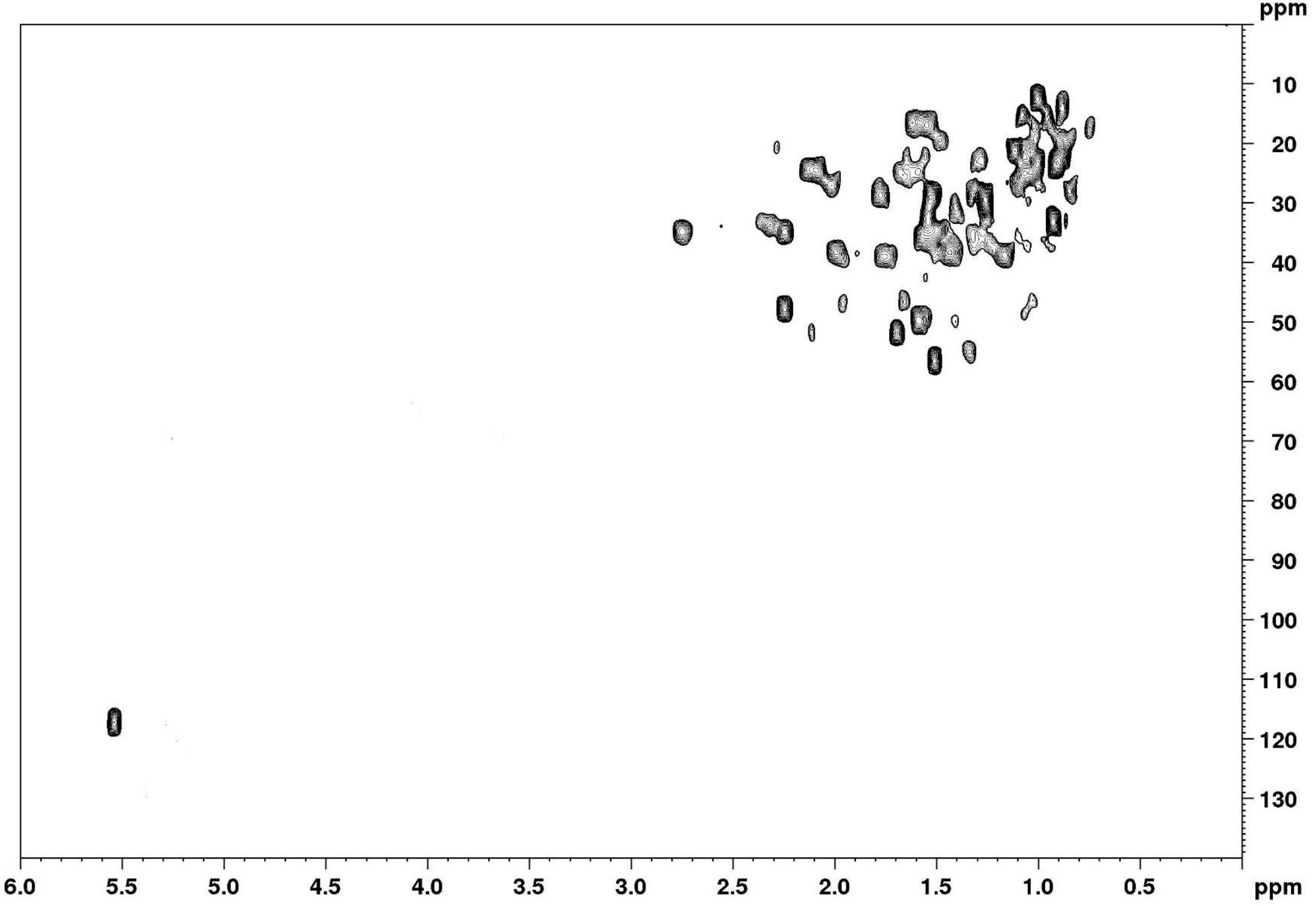


HSQC spectrum of acantholupenone (**1**) (600 MHz, $CDCl_3$).

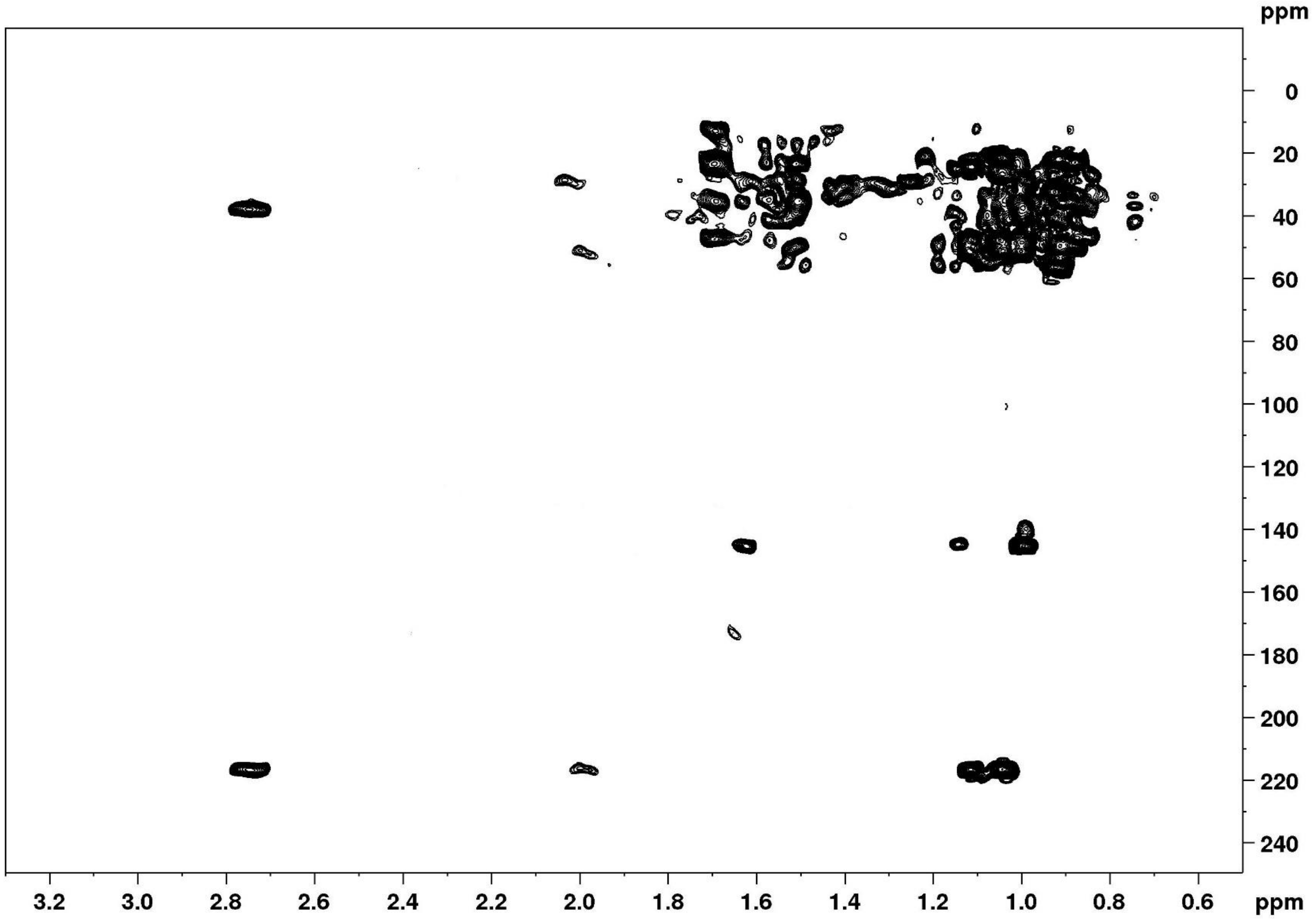


HMBC spectrum of acantholupenone (**1**) (600 MHz, $CDCl_3$).

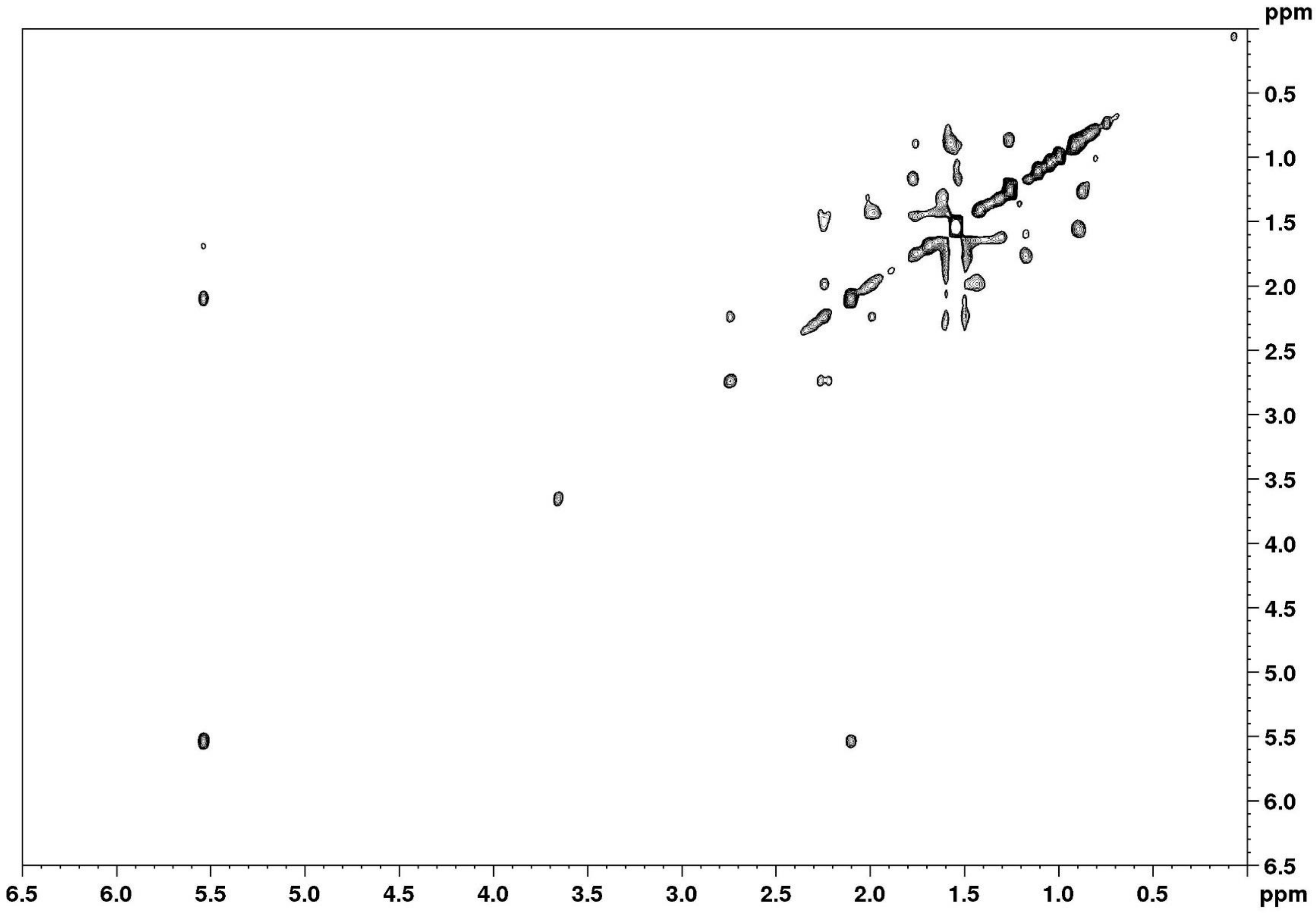


TOCSY spectrum of acantholupenone (**1**) (400 MHz, $CDCl_3$).

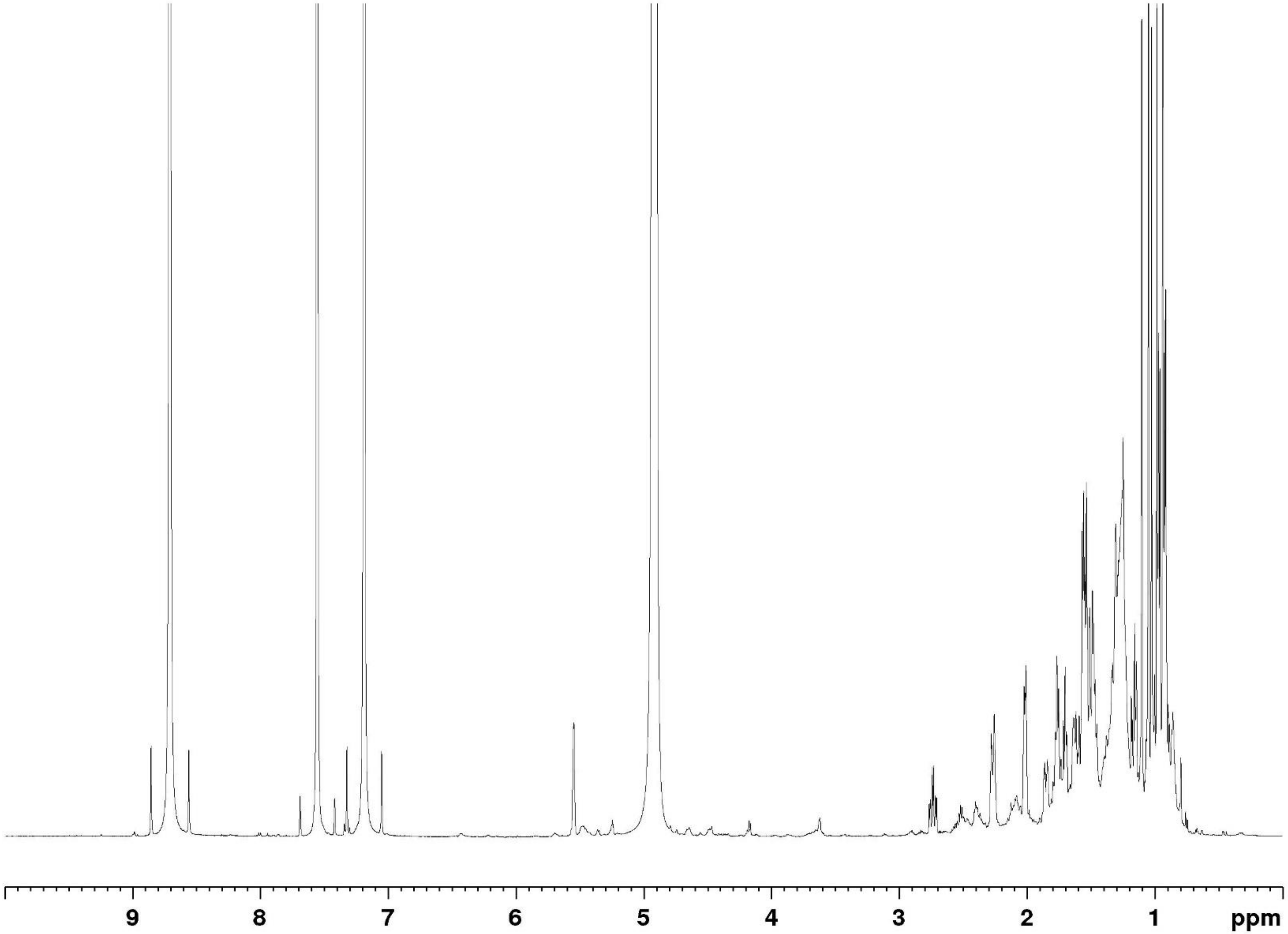


$^{1}$H NMR spectrum of acantholupenone (**1**) (600 MHz, $C_5D_5N$).

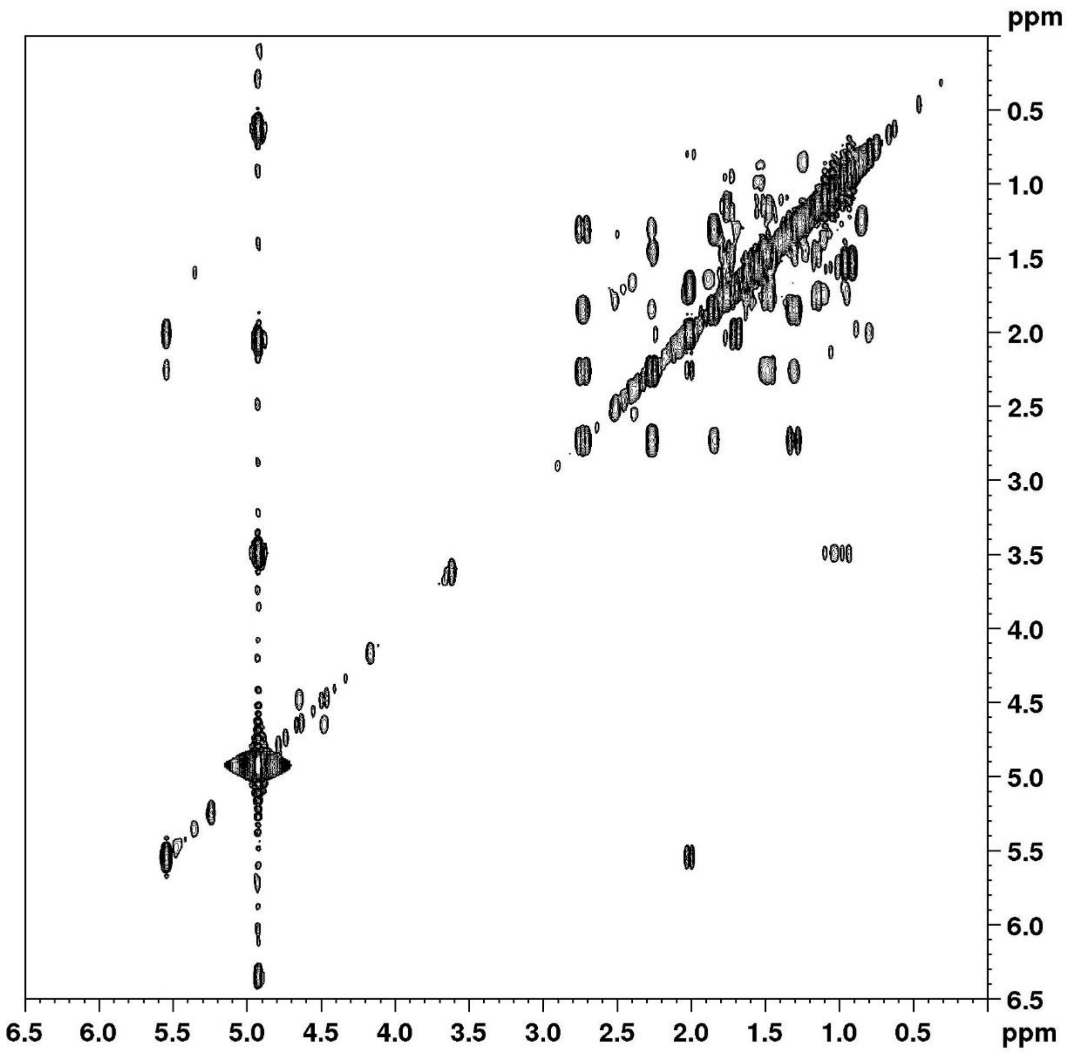


COSY spectrum of acantholupenone (**1**) (600 MHz, $C_5D_5N$).

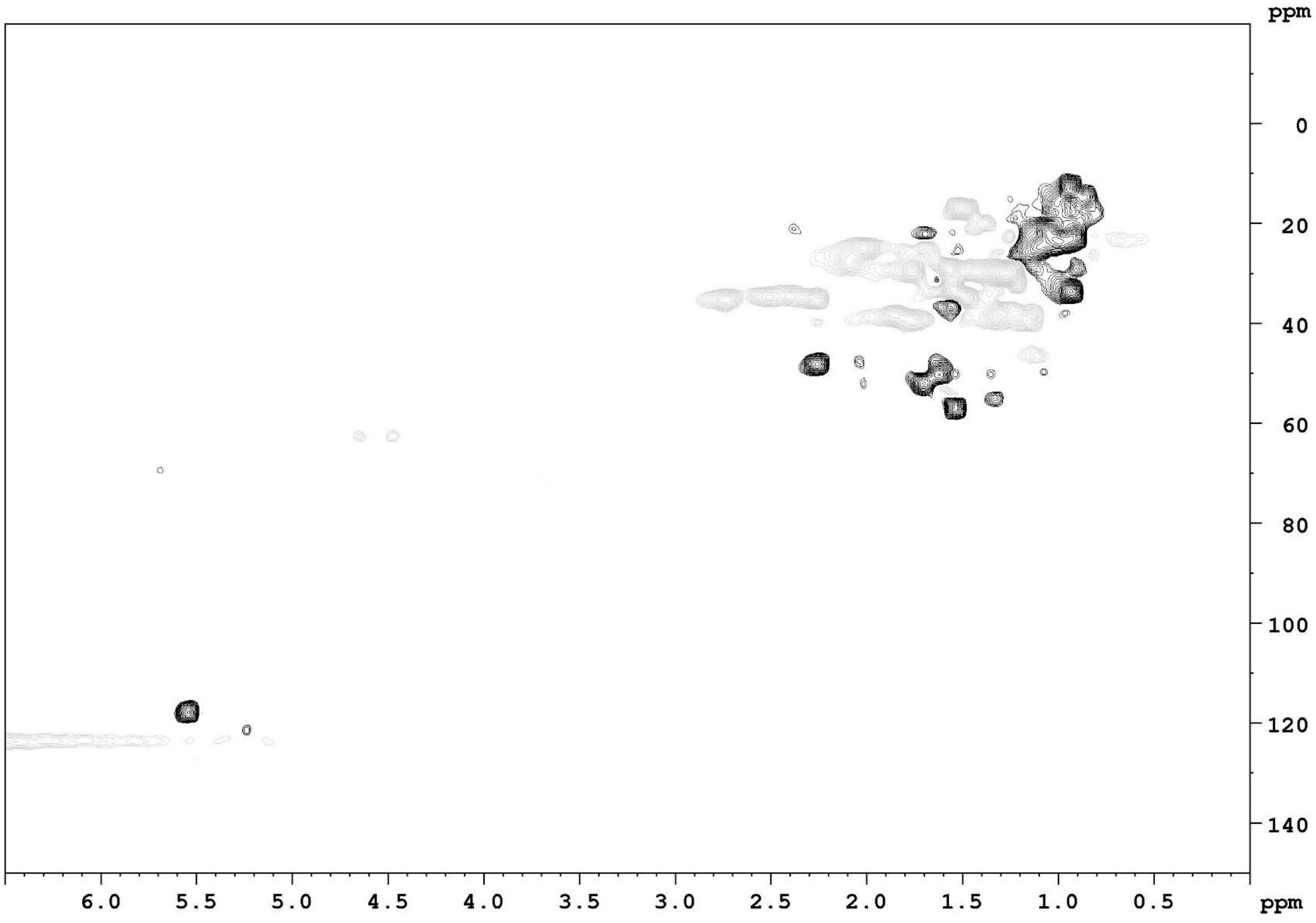


edHSQC spectrum of acantholupenone (**1**) (600 MHz, $C_5D_5N$).

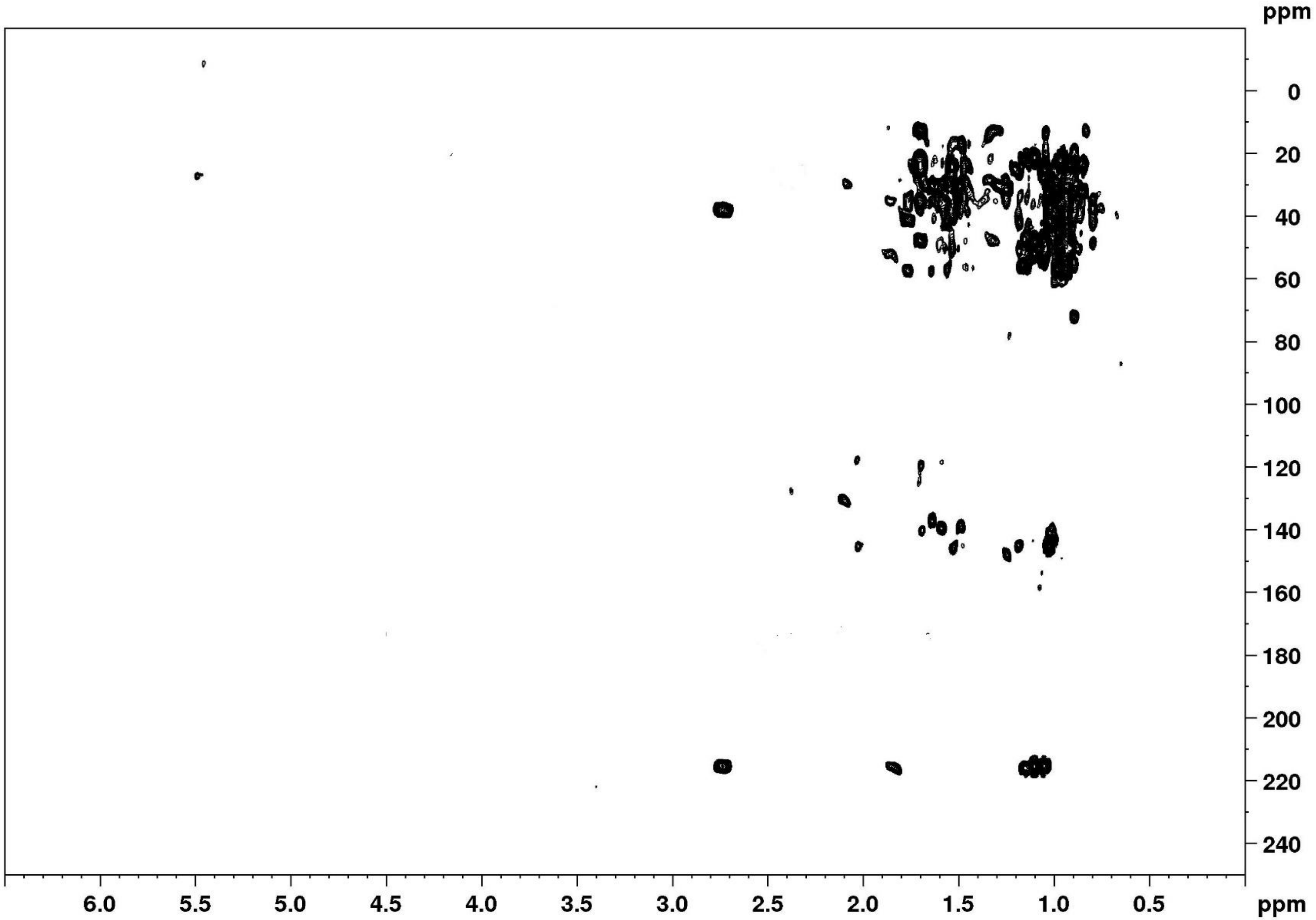


HMBC spectrum of acantholupenone (**1**) (600 MHz, $C_5D_5N$).

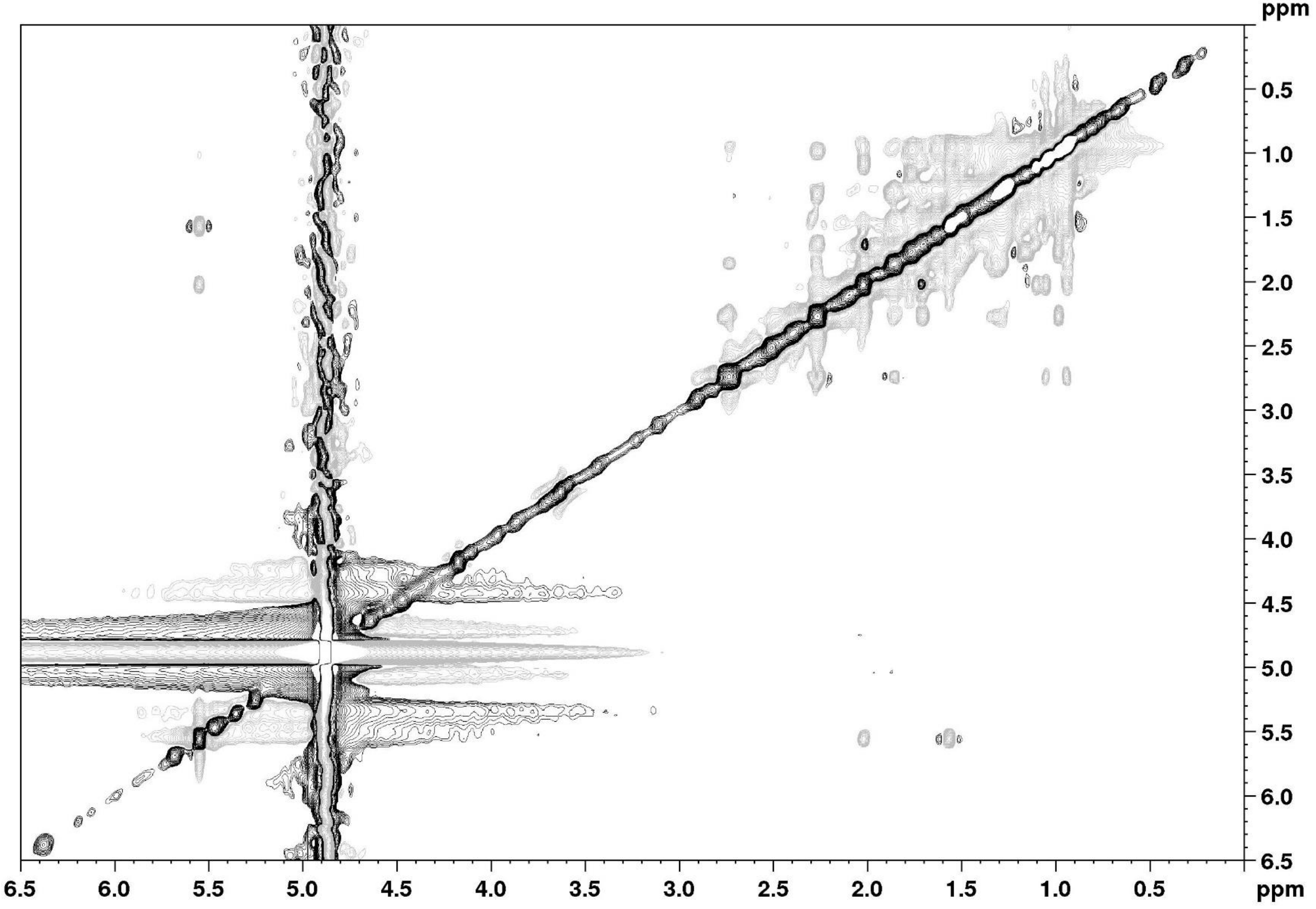


NOESY spectrum of acantholupenone (**1**) (400 MHz, $C_5D_5N$).

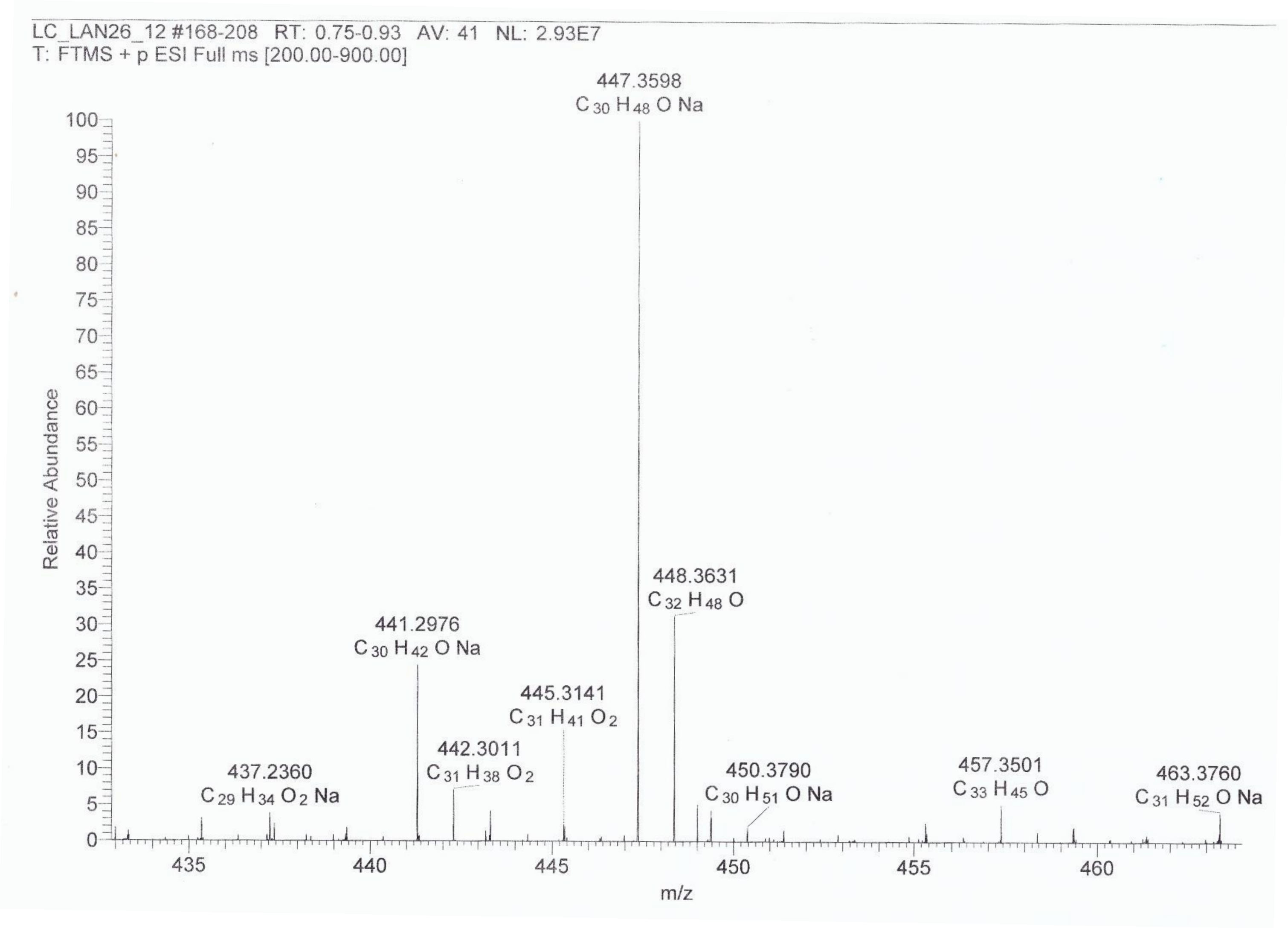


HRESIMS spectrum of acantholupenone (**1**).

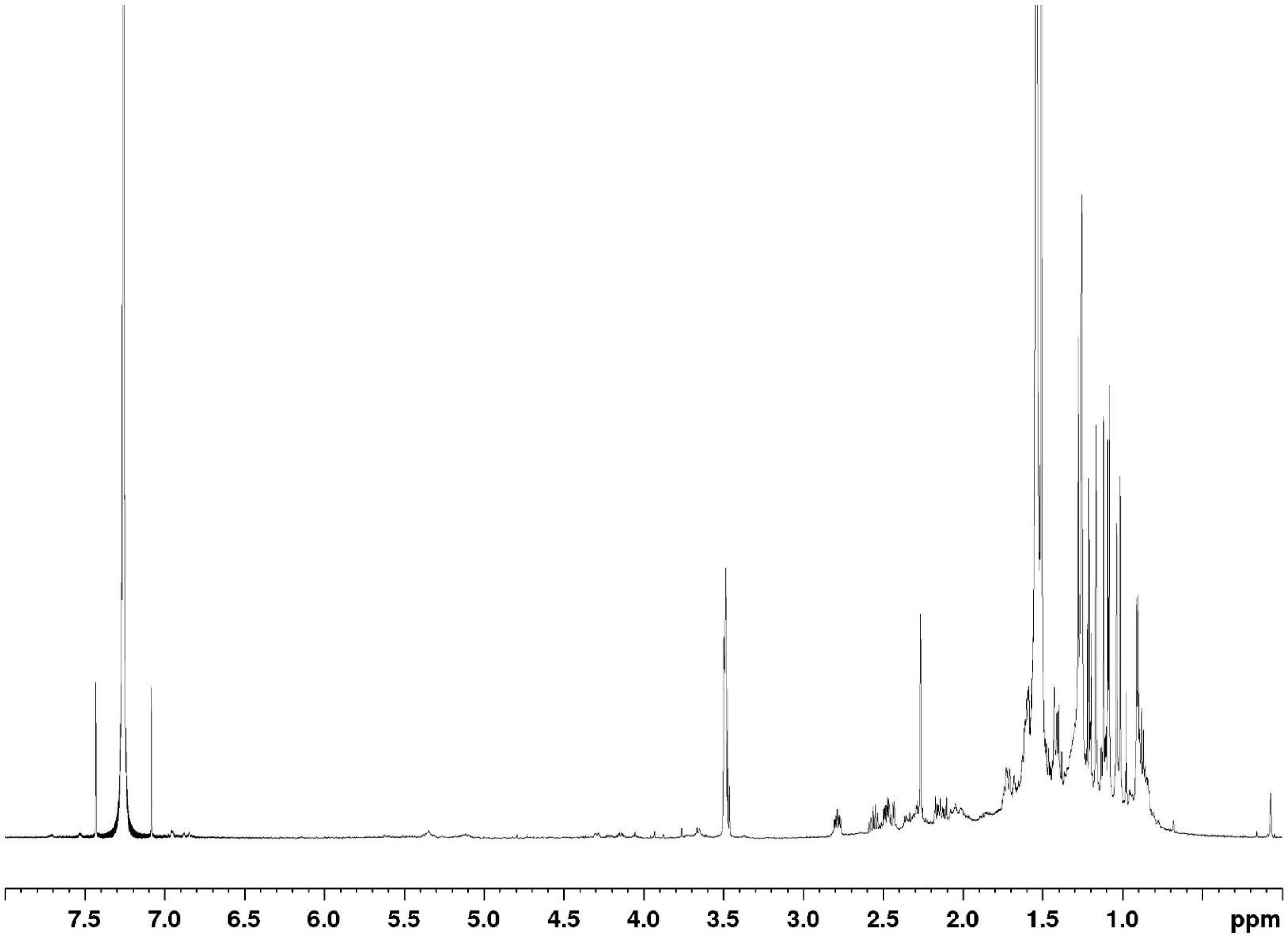


$^{1}$H NMR spectrum of acanthobauerendione (**2**) (600 MHz, $CDCl_3$).

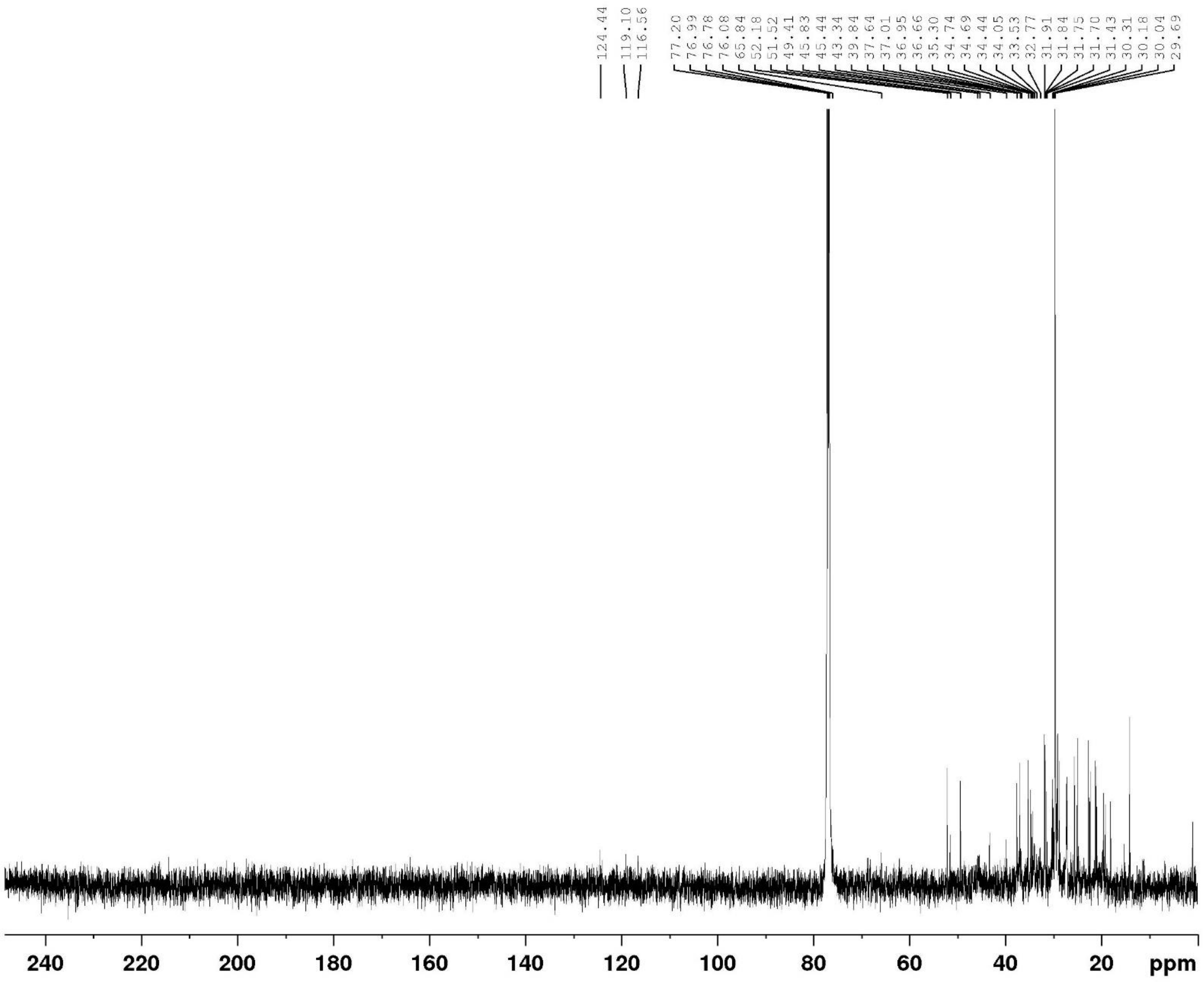


$^{13}C$ NMR spectrum of acanthobauerendione (**2**) (150 MHz, $CDCl_3$).

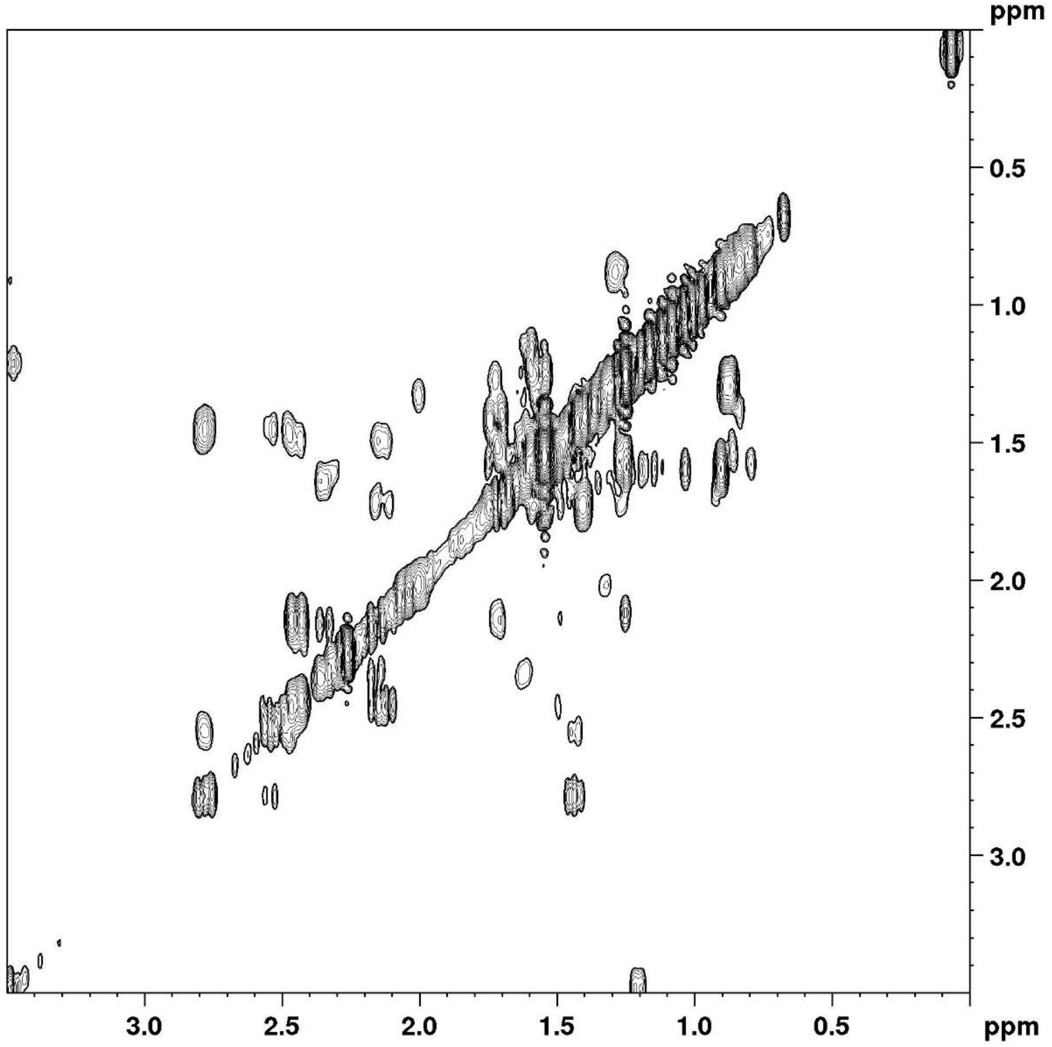


COSY spectrum of acanthobauerendione (**2**) (600 MHz, $CDCl_3$).

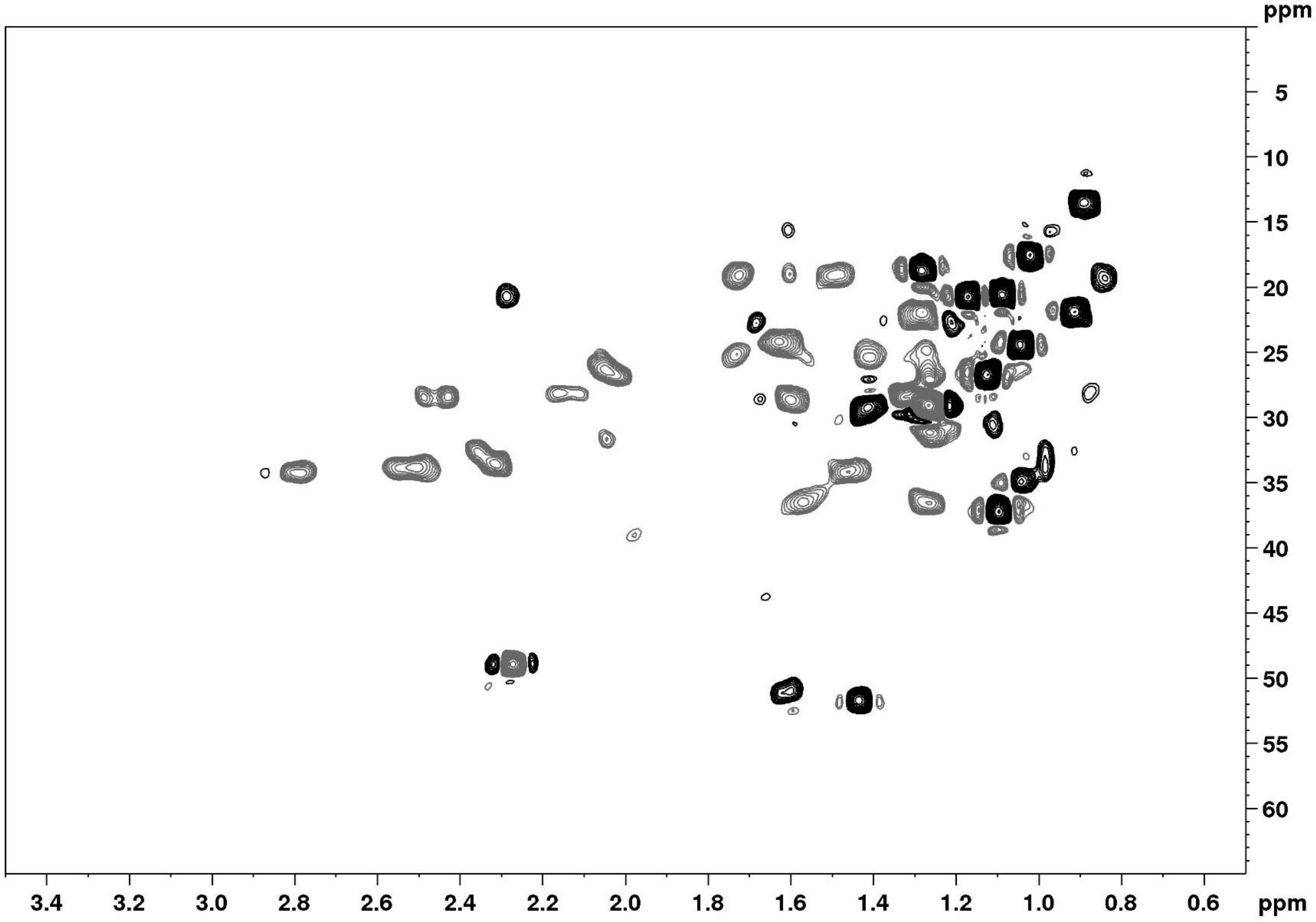


edHSQC spectrum of acanthobauerendione (**2**) (400 MHz, $CDCl_3$).

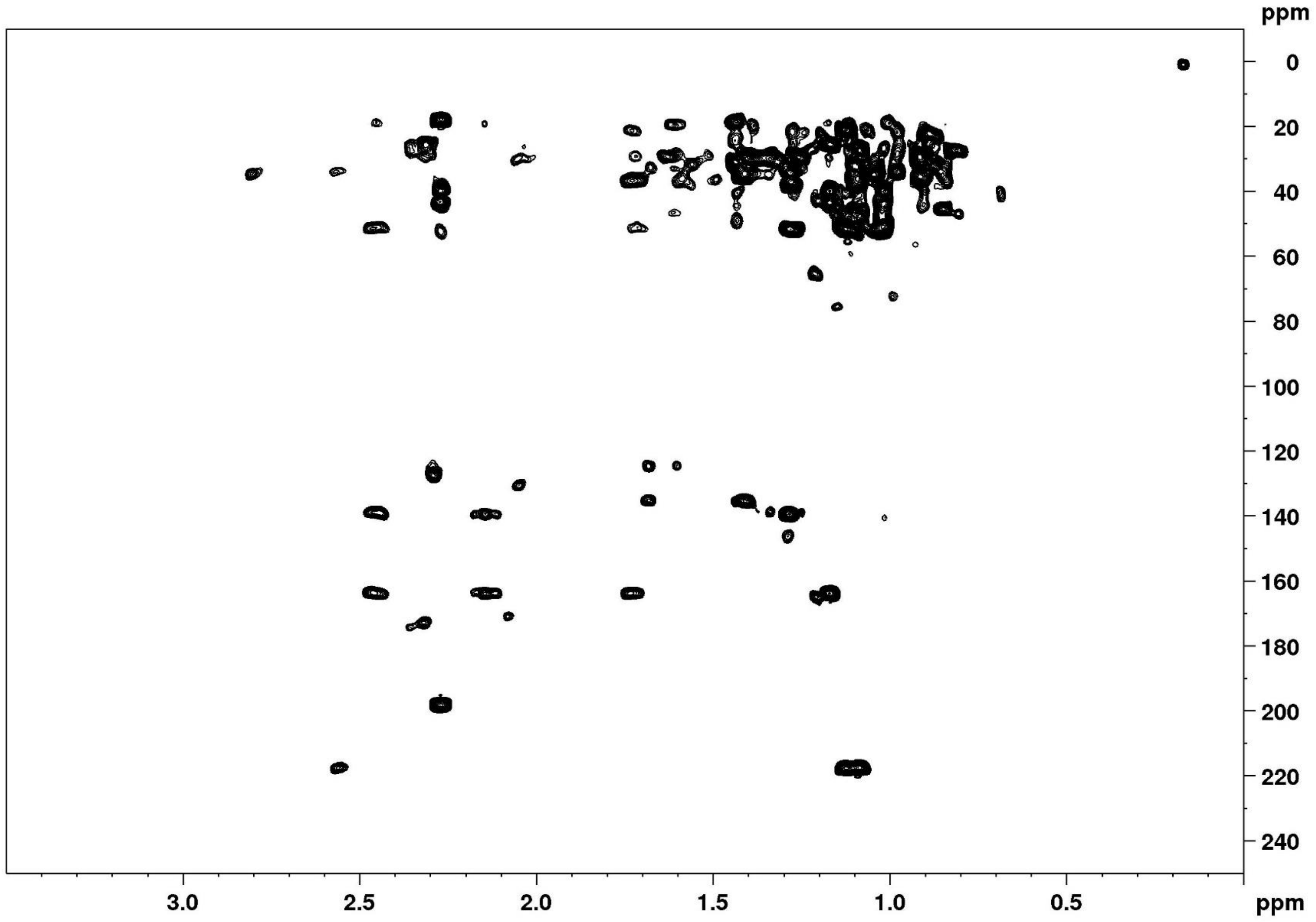


HMBC spectrum of acanthobauerendione (**2**) (600 MHz, $CDCl_3$).

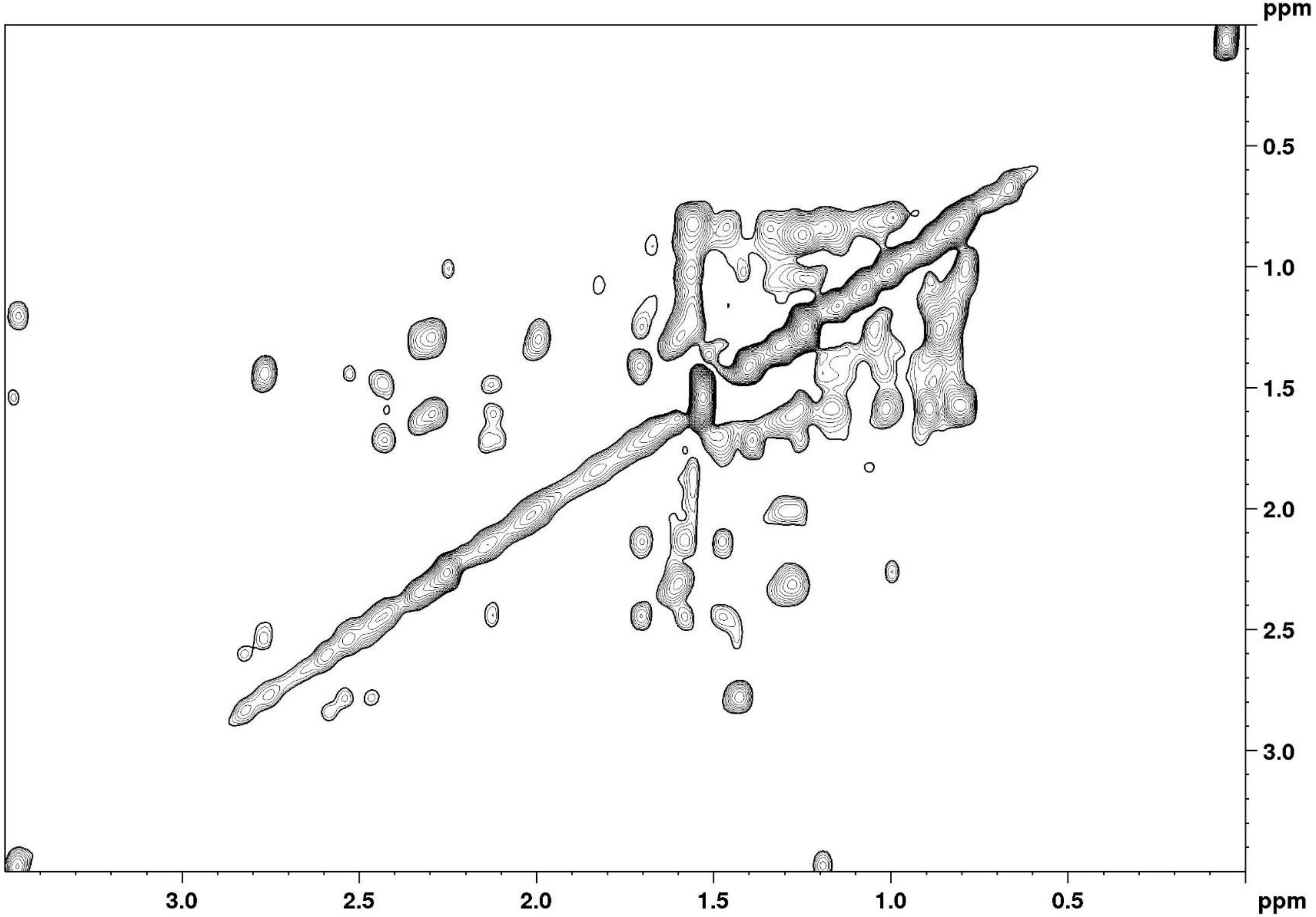


TOCSY spectrum of acanthobauerendione (**2**) (600 MHz, $CDCl_3$).

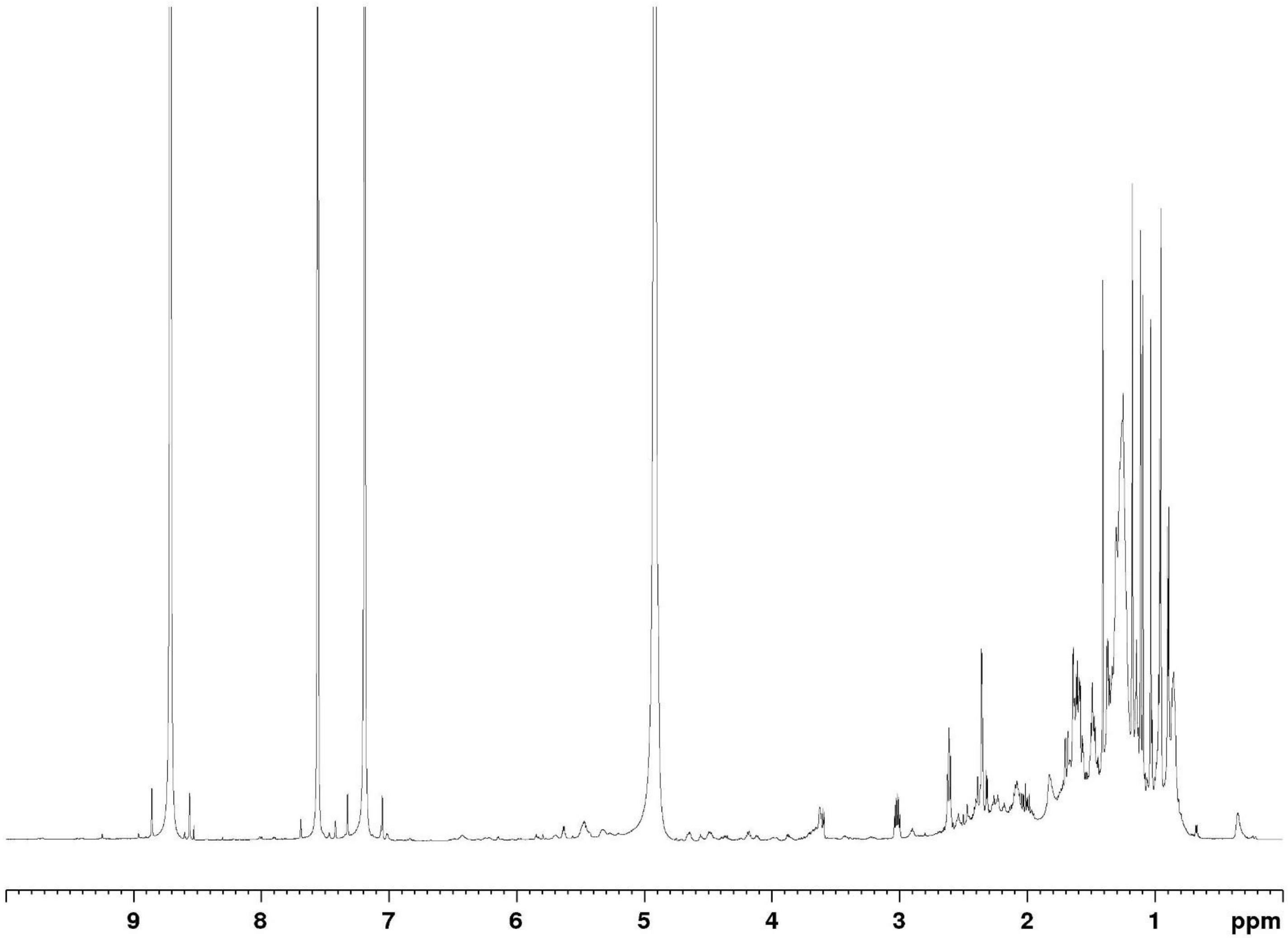


$^{1}$H NMR spectrum of acanthobauerendione (**2**) (600 MHz, $C_5D_5N$).

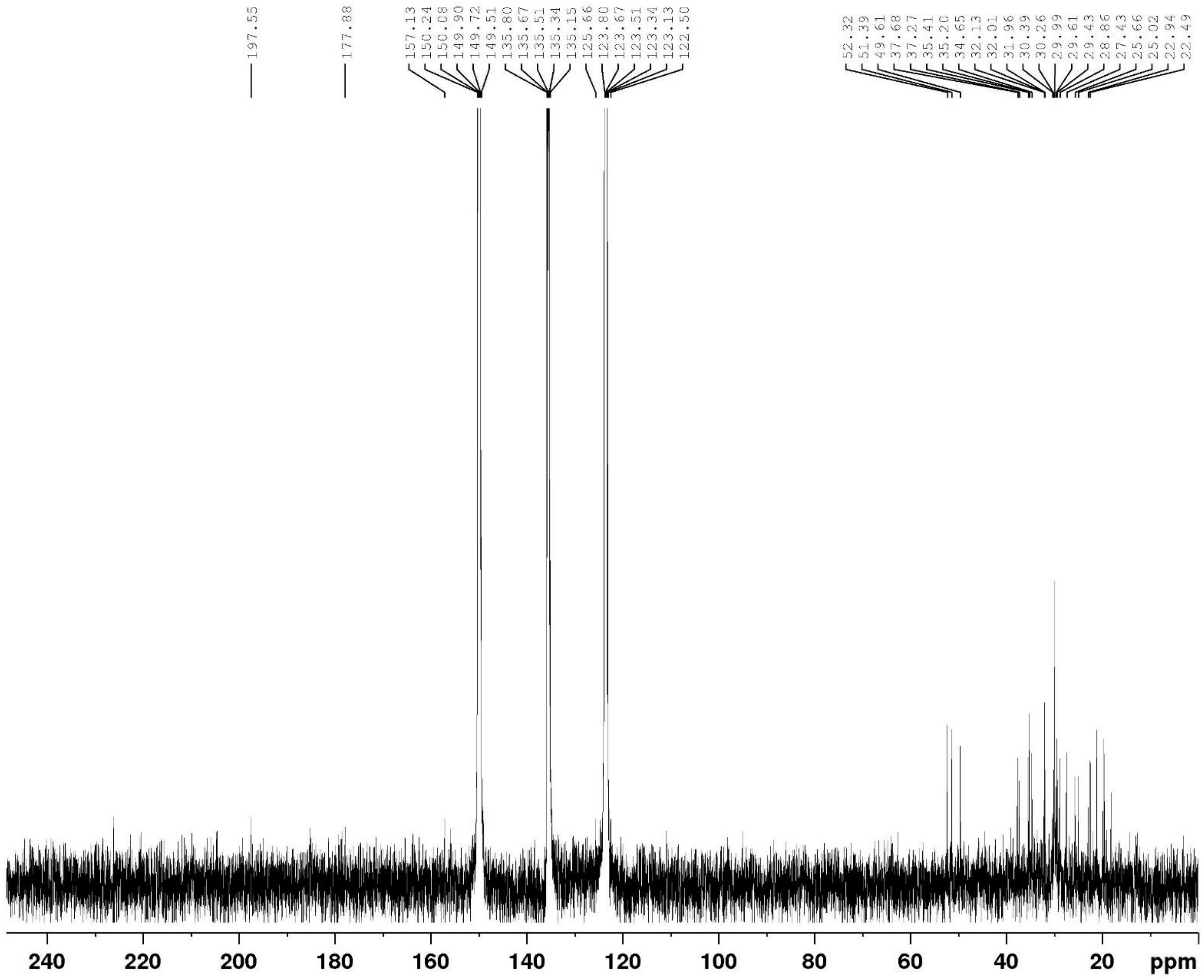


$^{13}$C NMR spectrum of acanthobauerendione (**2**) (150 MHz, $C_5D_5N$).

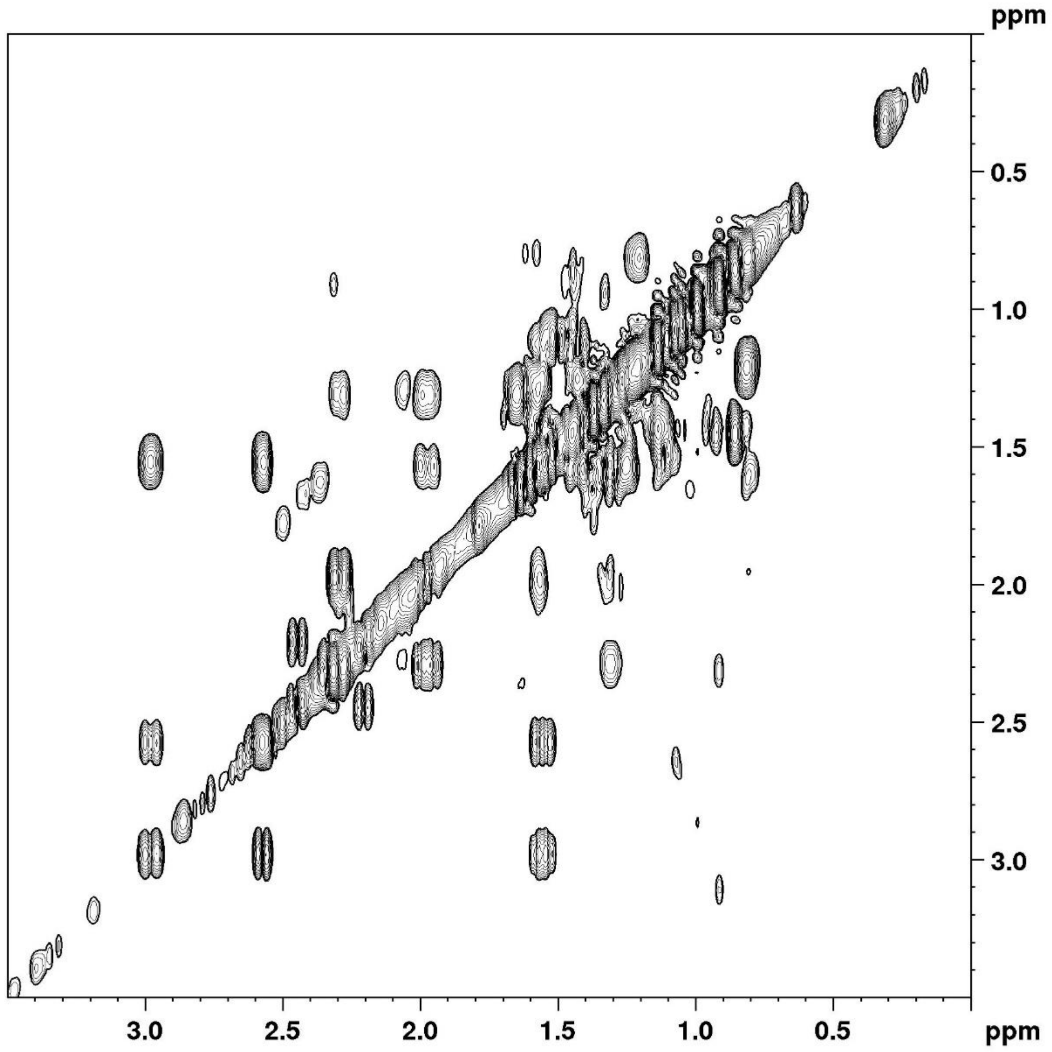


COSY spectrum of acanthobauerendione (**2**) (600 MHz, $C_5D_5N$).

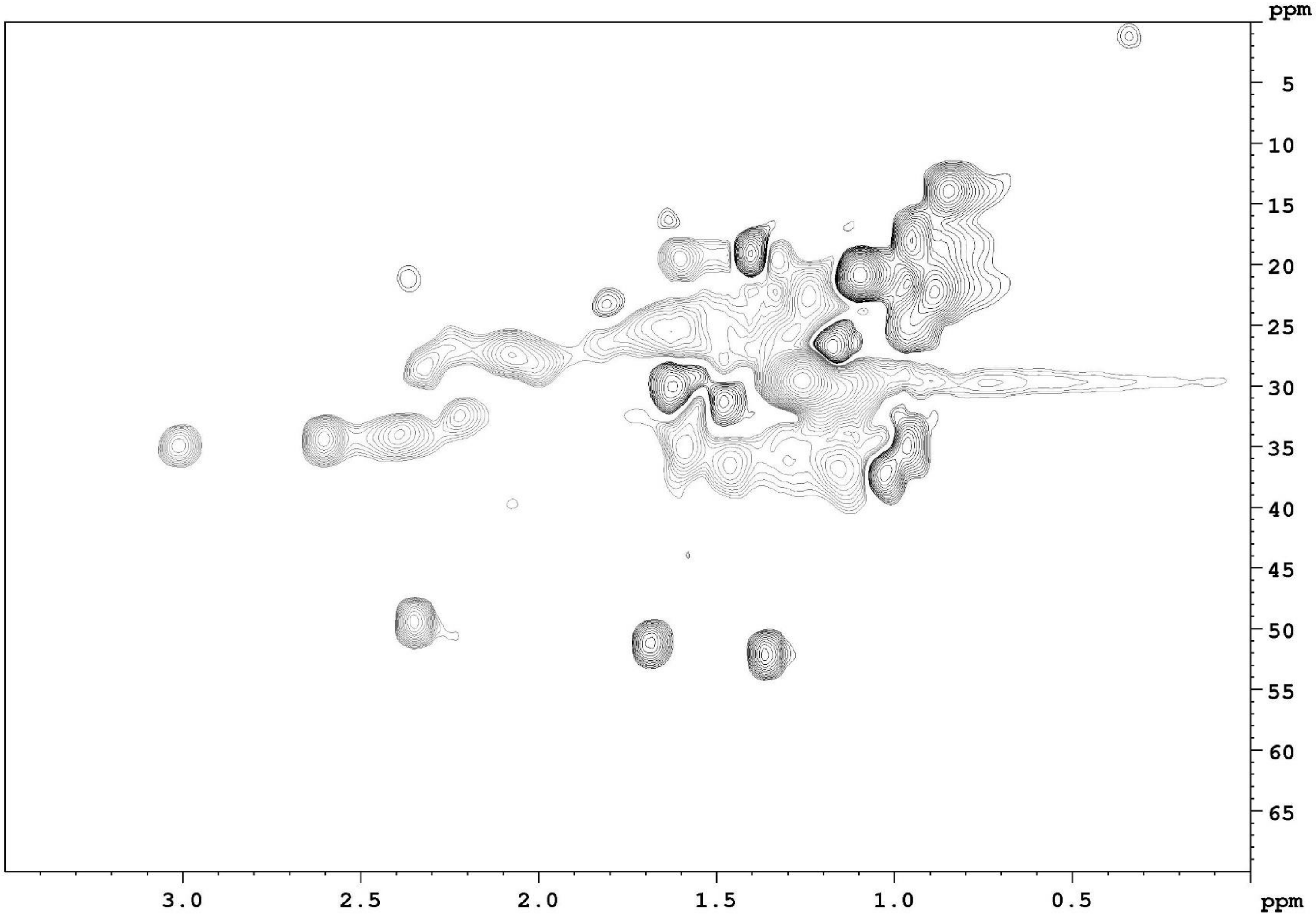


edHSQC spectrum of acanthobauerendione (**2**) (600 MHz, $C_5D_5N$).

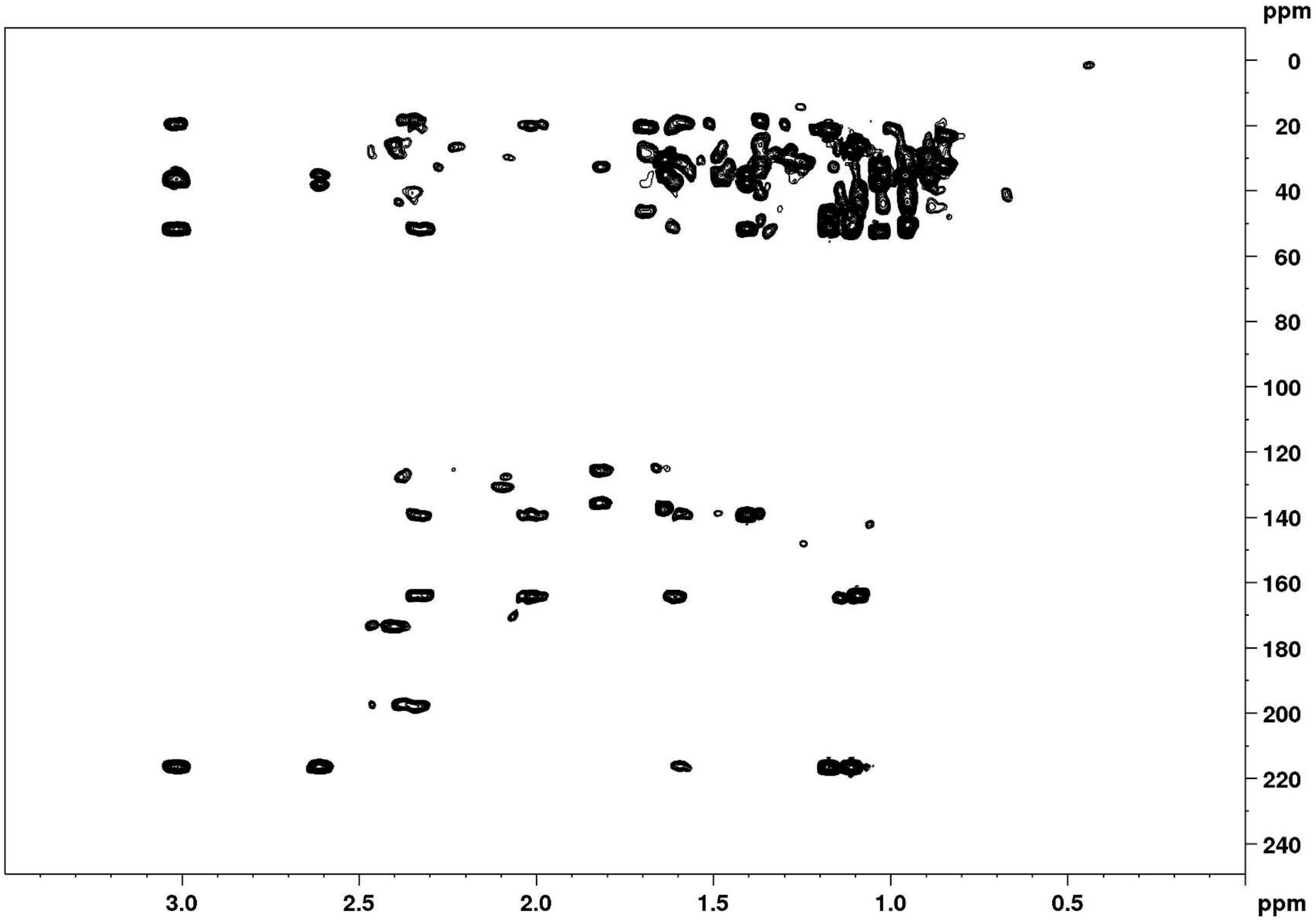


HMBC spectrum of acanthobauerendione (**2**) (600 MHz, $C_5D_5N$).

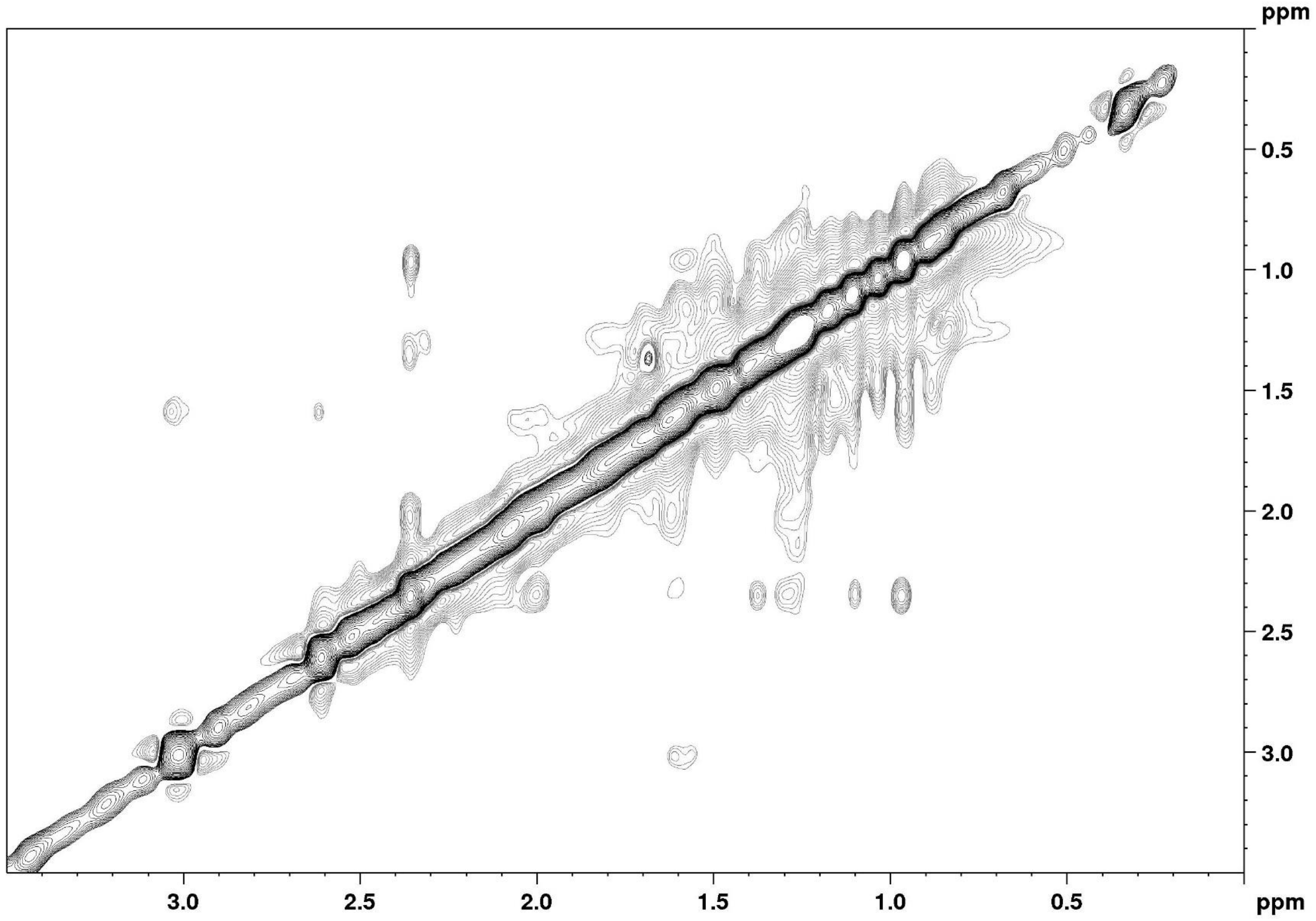


NOESY spectrum of acanthobauerendione (**2**) (400 MHz, $C_5D_5N$)

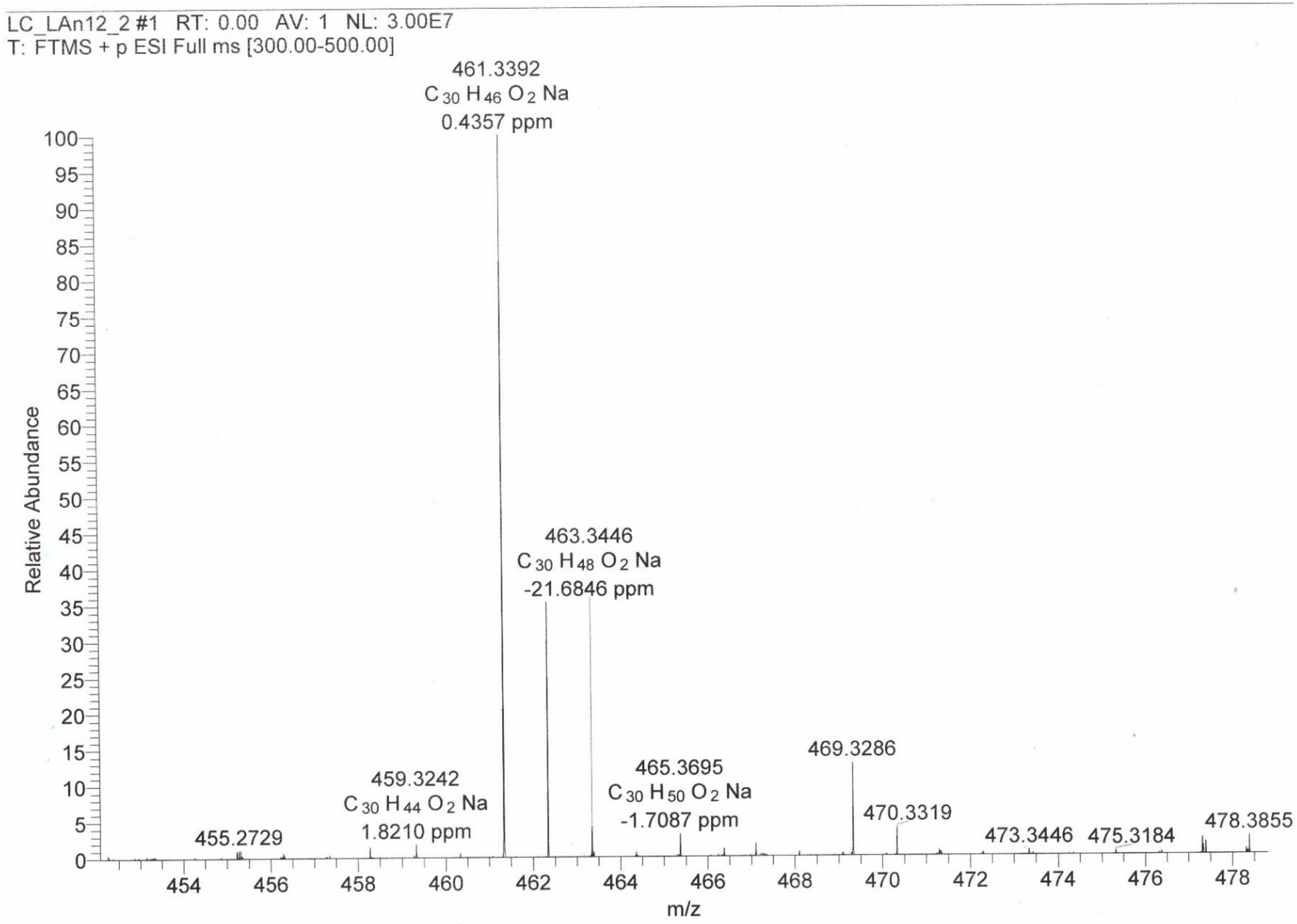


HRESIMS spectrum of acanthobauerendione (**2**).

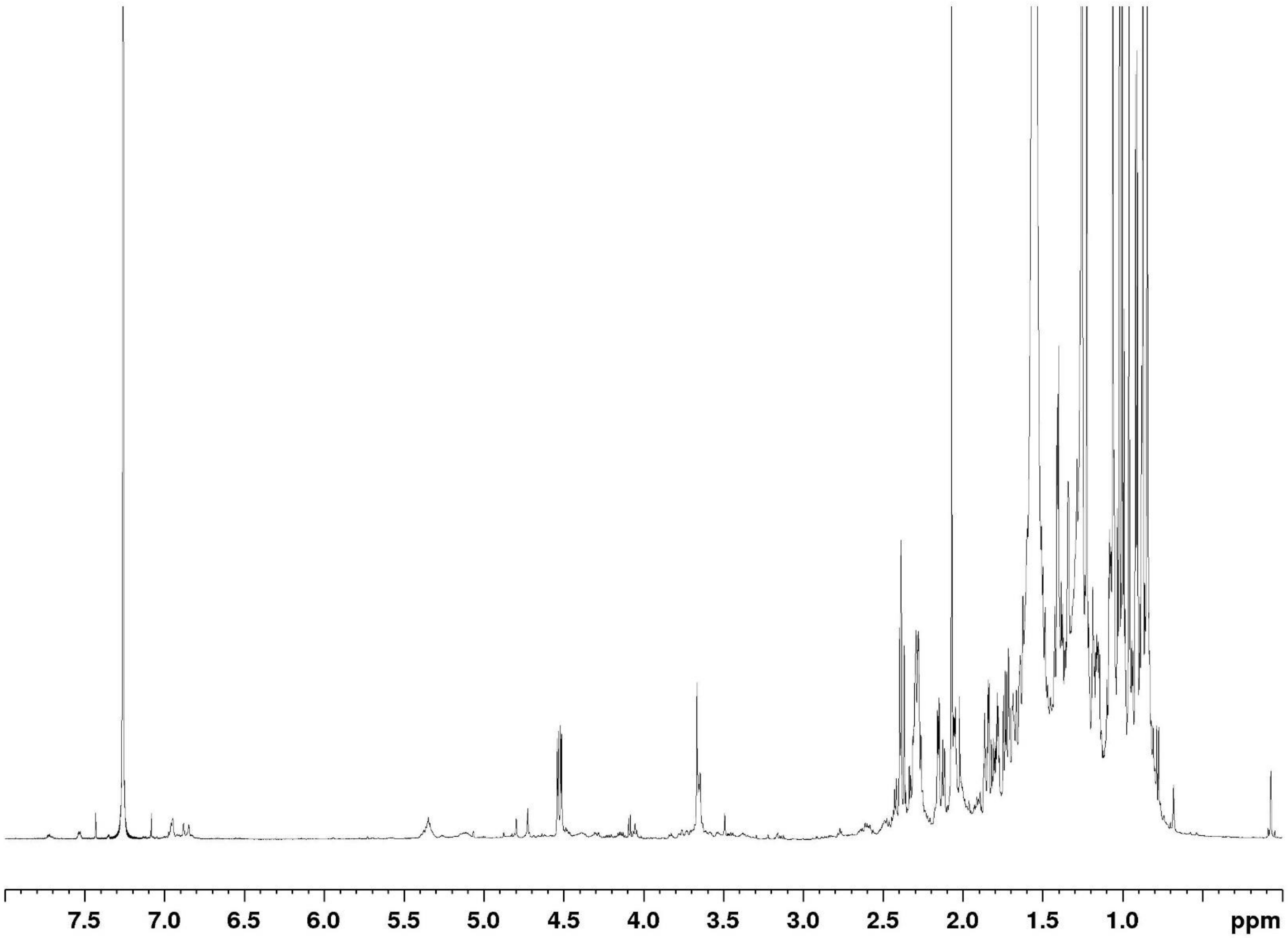


$^{1}$H NMR spectrum of acanthobauerenone (**3**) (600 MHz, $CDCl_3$).

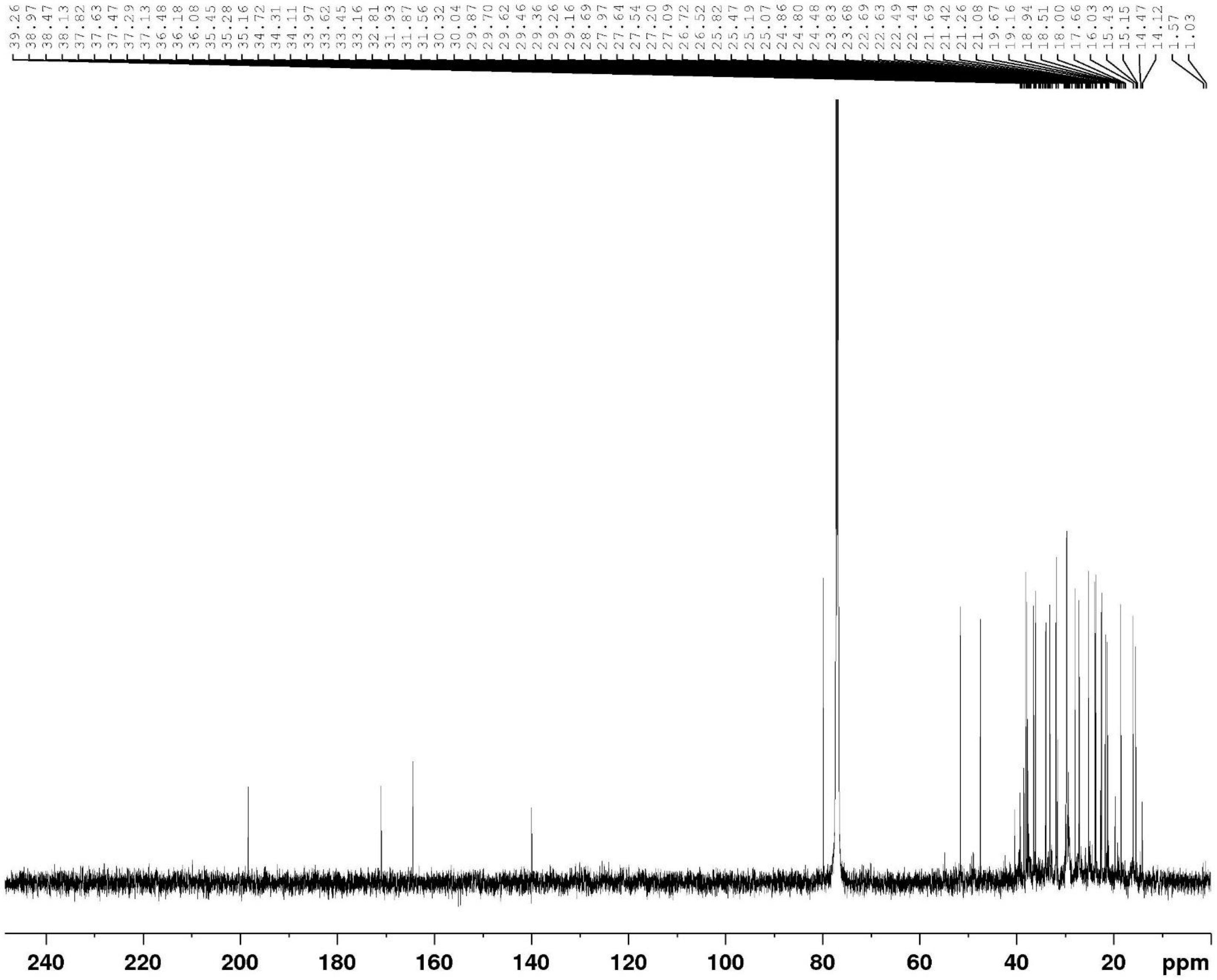


$^{13}$C NMR spectrum of acanthobauerenone (**3**) (150 MHz, $CDCl_3$).

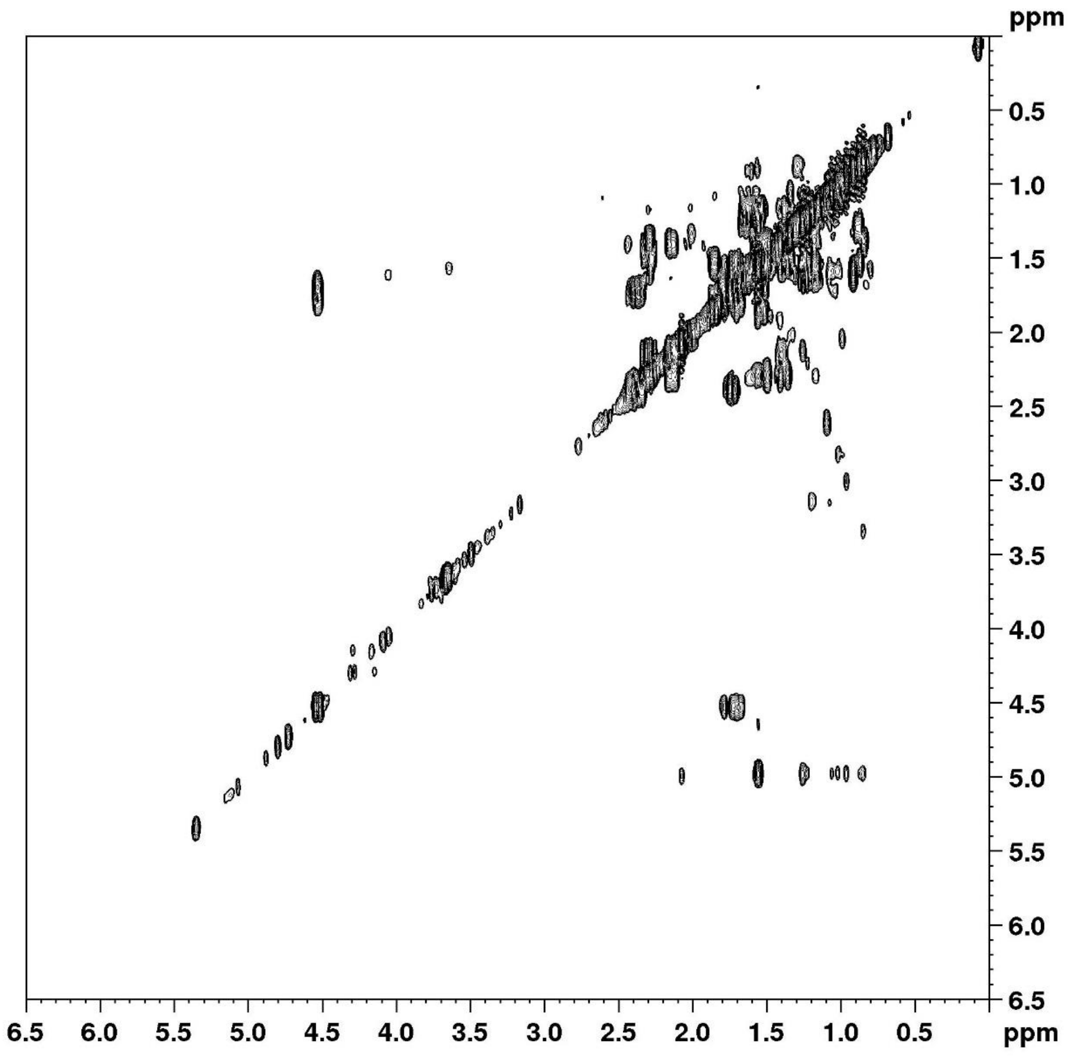


COSY spectrum of acanthobauerenone (**3**) (600 MHz, $CDCl_3$).

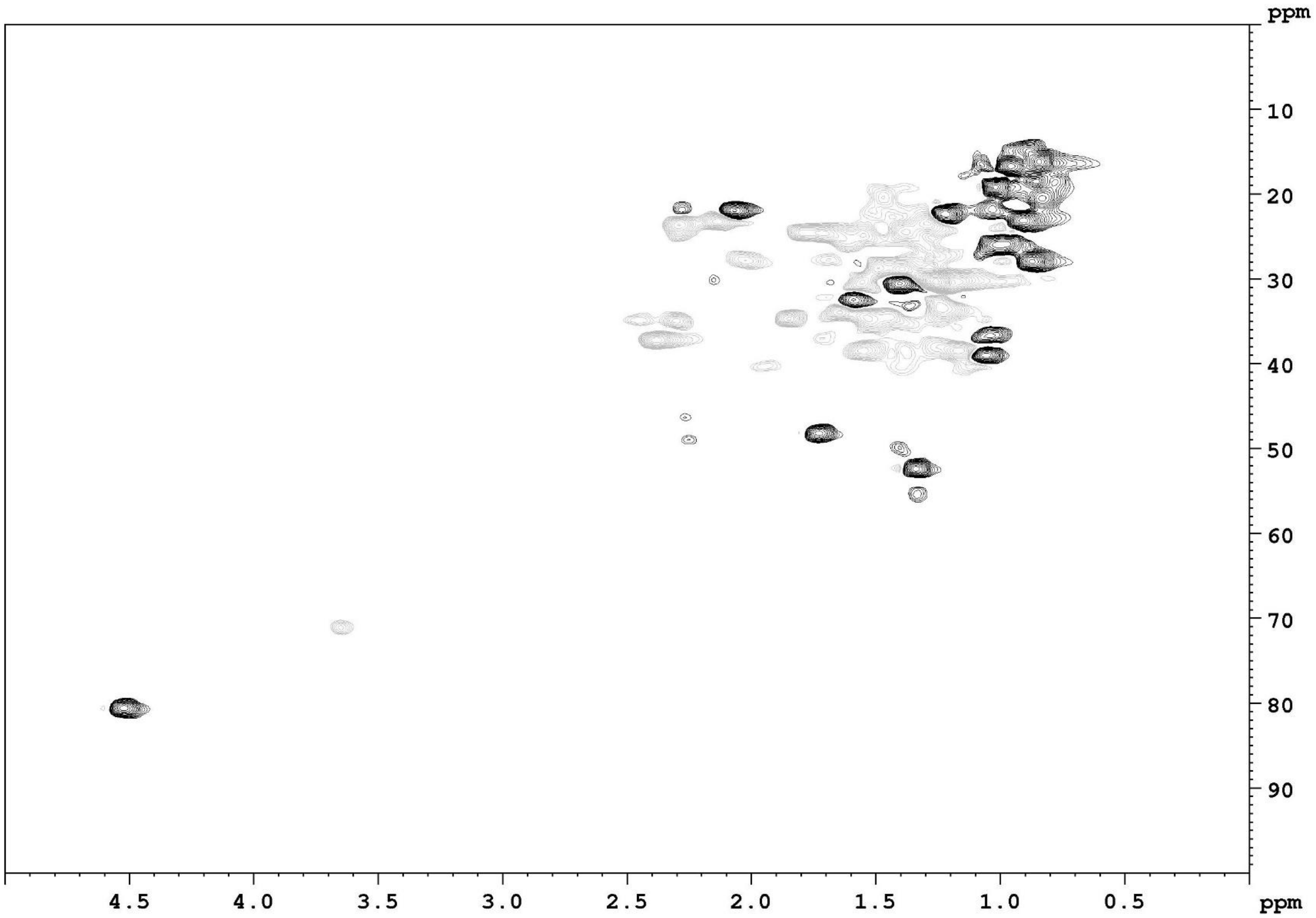


edHSQC spectrum of acanthobauerenone (**3**) (600 MHz, $CDCl_3$).

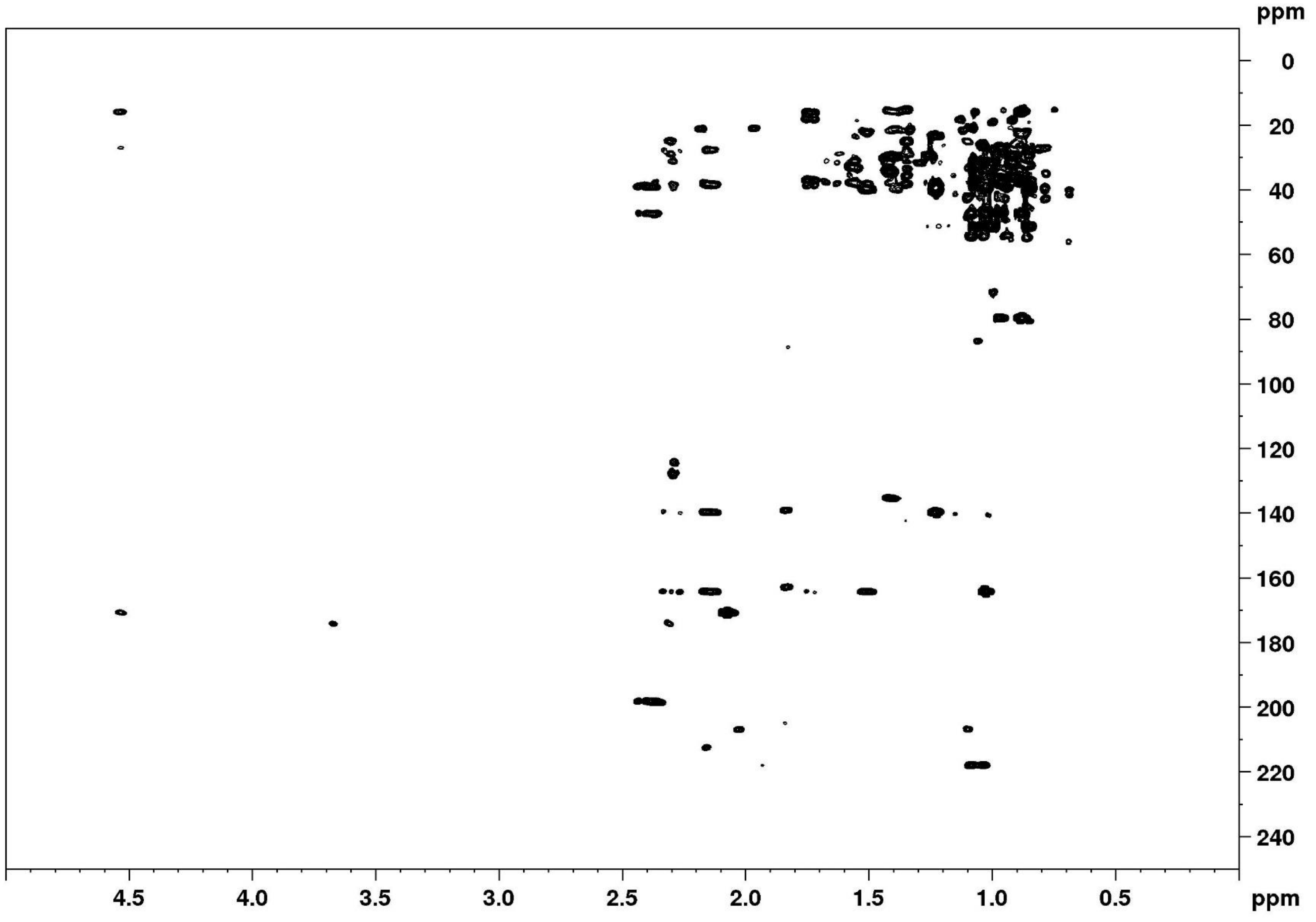


HMBC spectrum of acanthobauerenone (**3**) (600 MHz, $CDCl_3$).

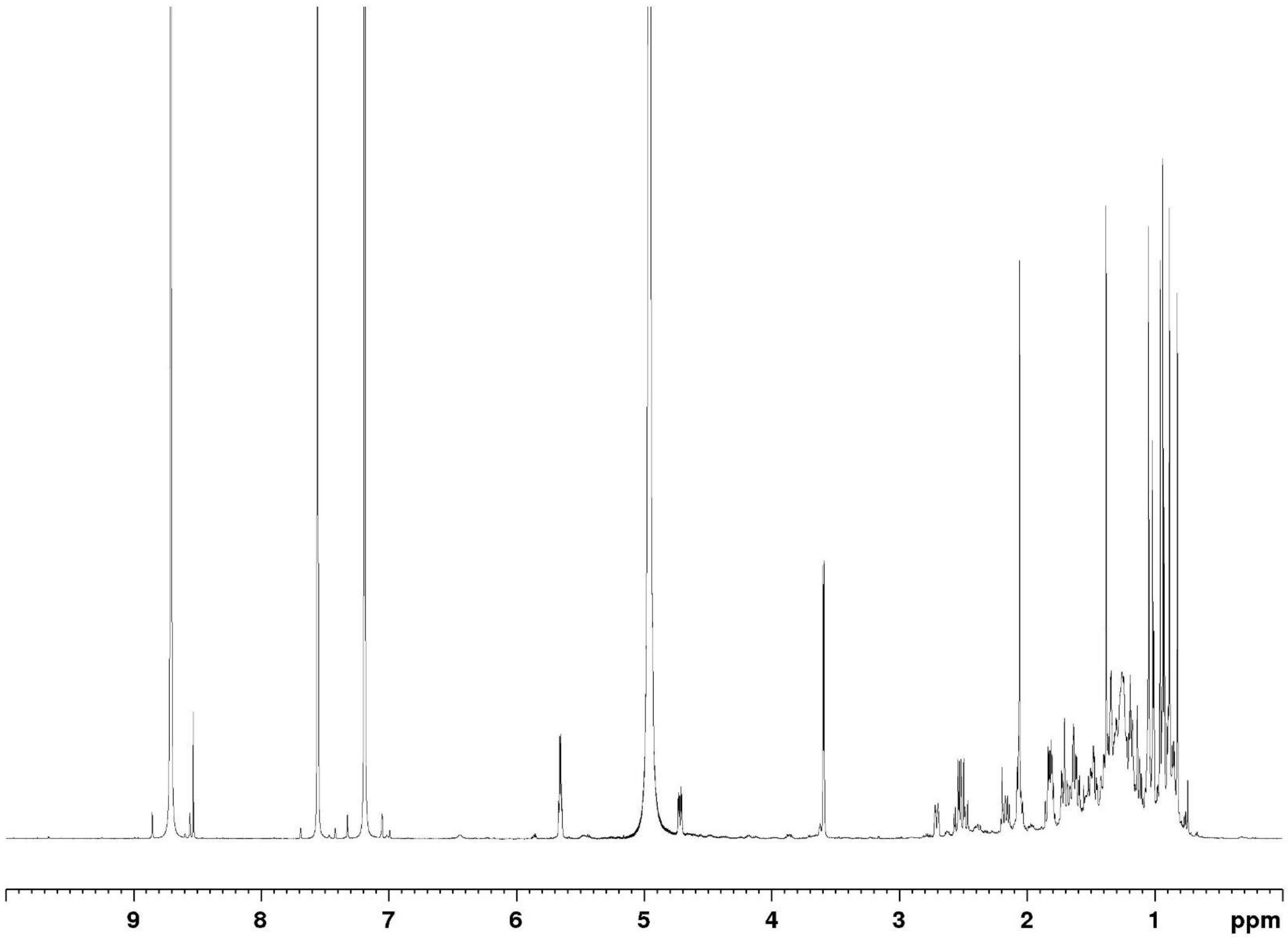


$^{1}$H NMR spectrum of acanthobauerenone (**3**) (600 MHz, $C_5D_5N$).

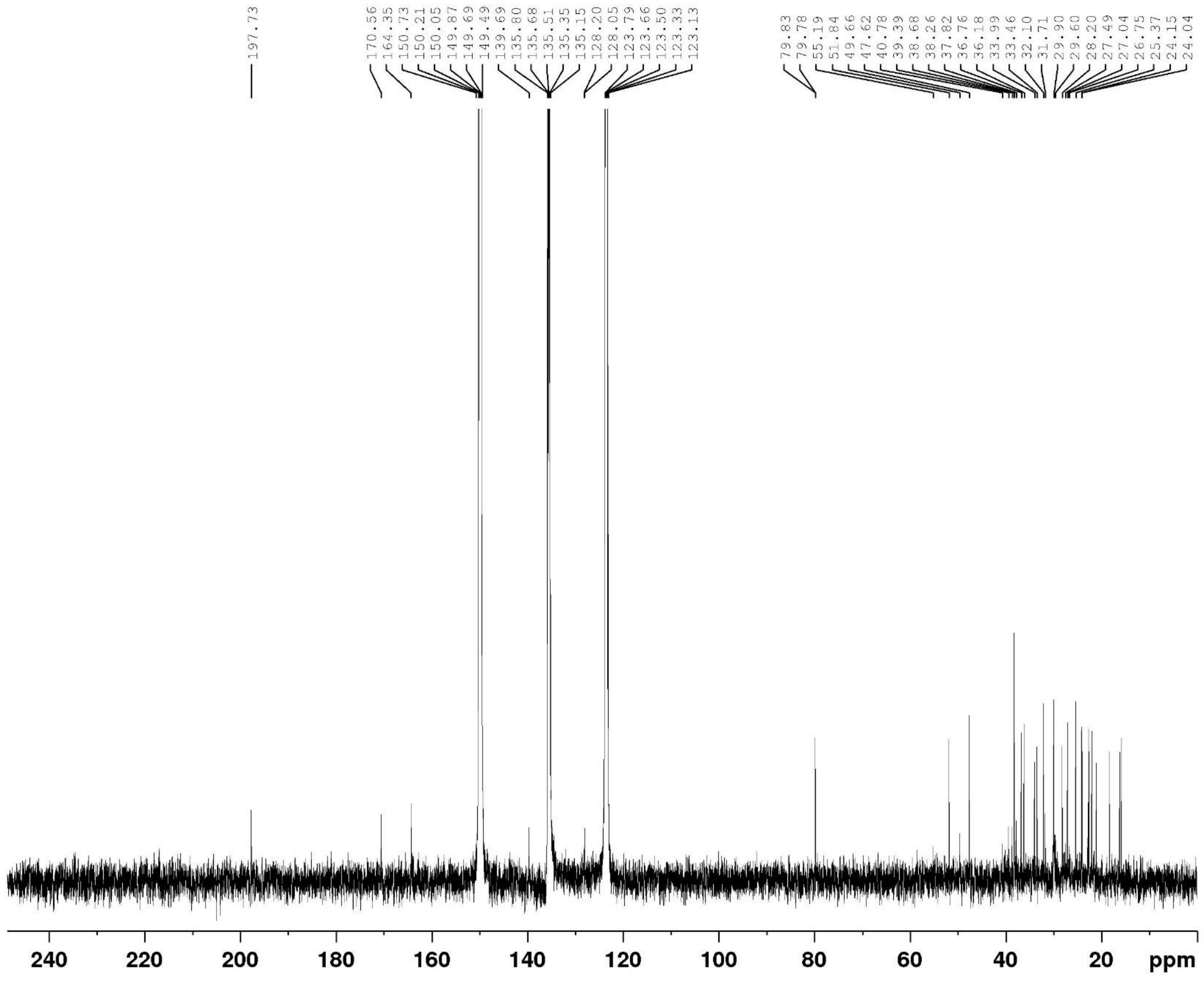


$^{13}$C NMR spectrum of acanthobauerenone (**3**) (150 MHz, $C_5D_5N$).

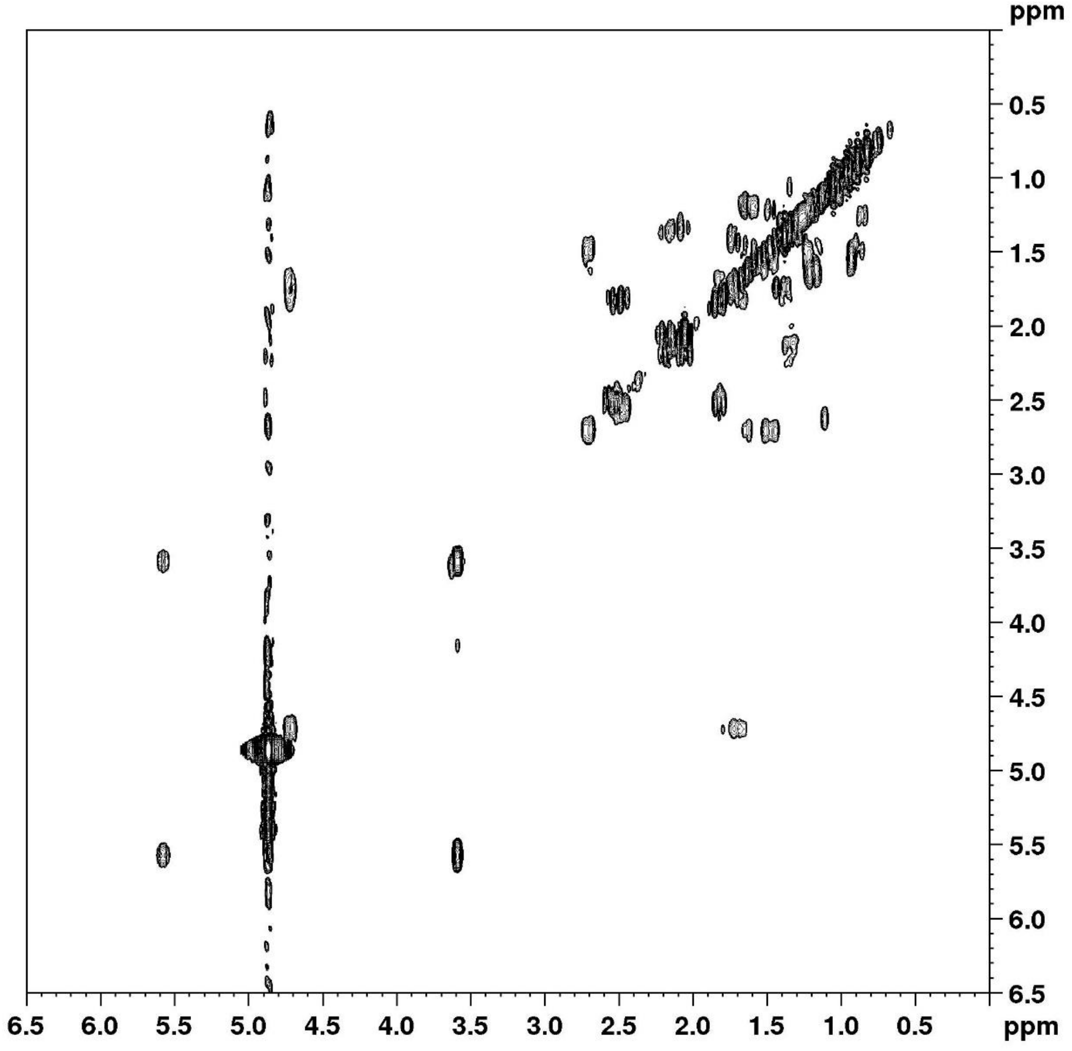


COSY spectrum of acanthobauerenone (**3**) (600 MHz, $C_5D_5N$).

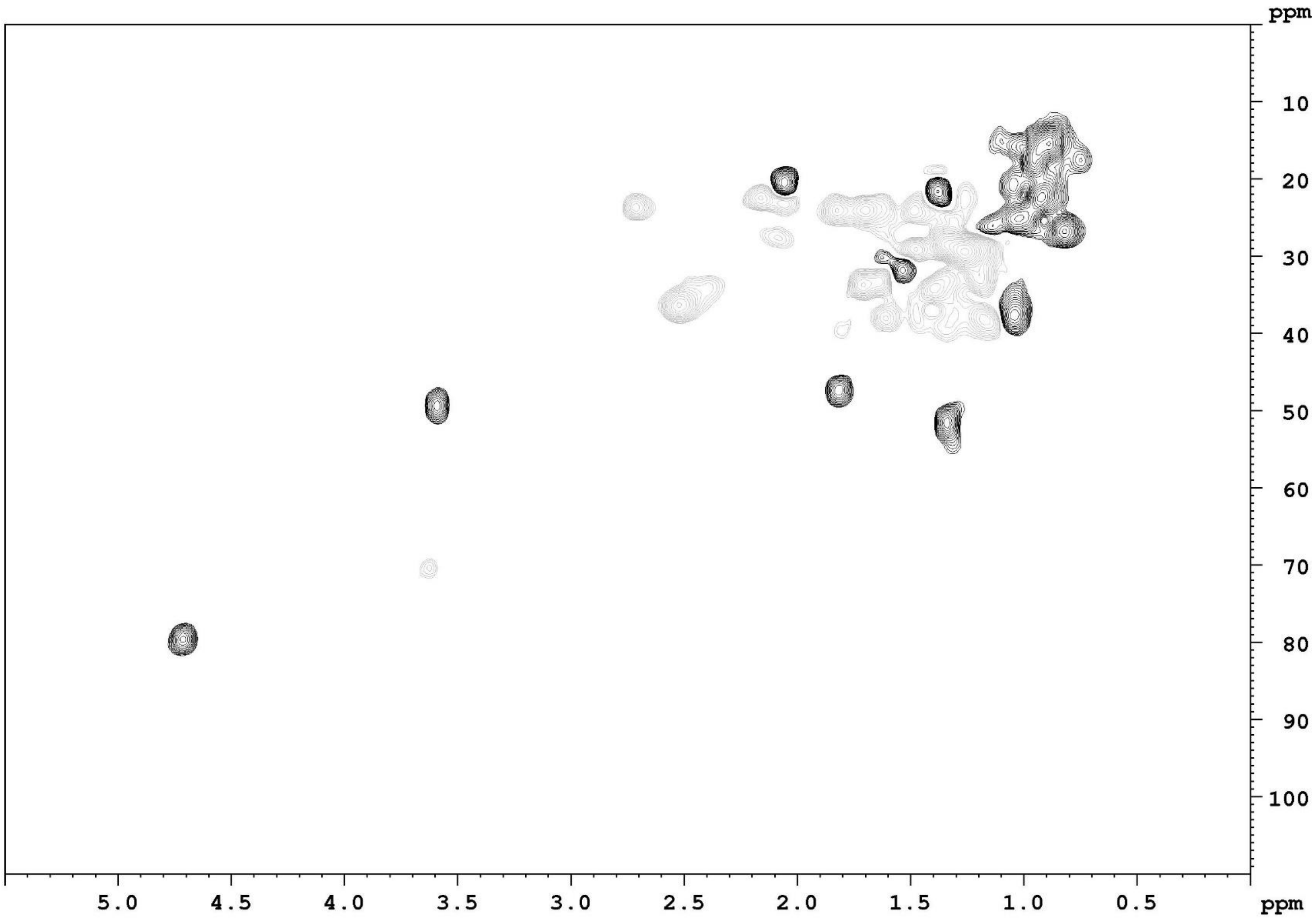


edHSQC spectrum of acanthobauerenone (**3**) (600 MHz, $C_5D_5N$).

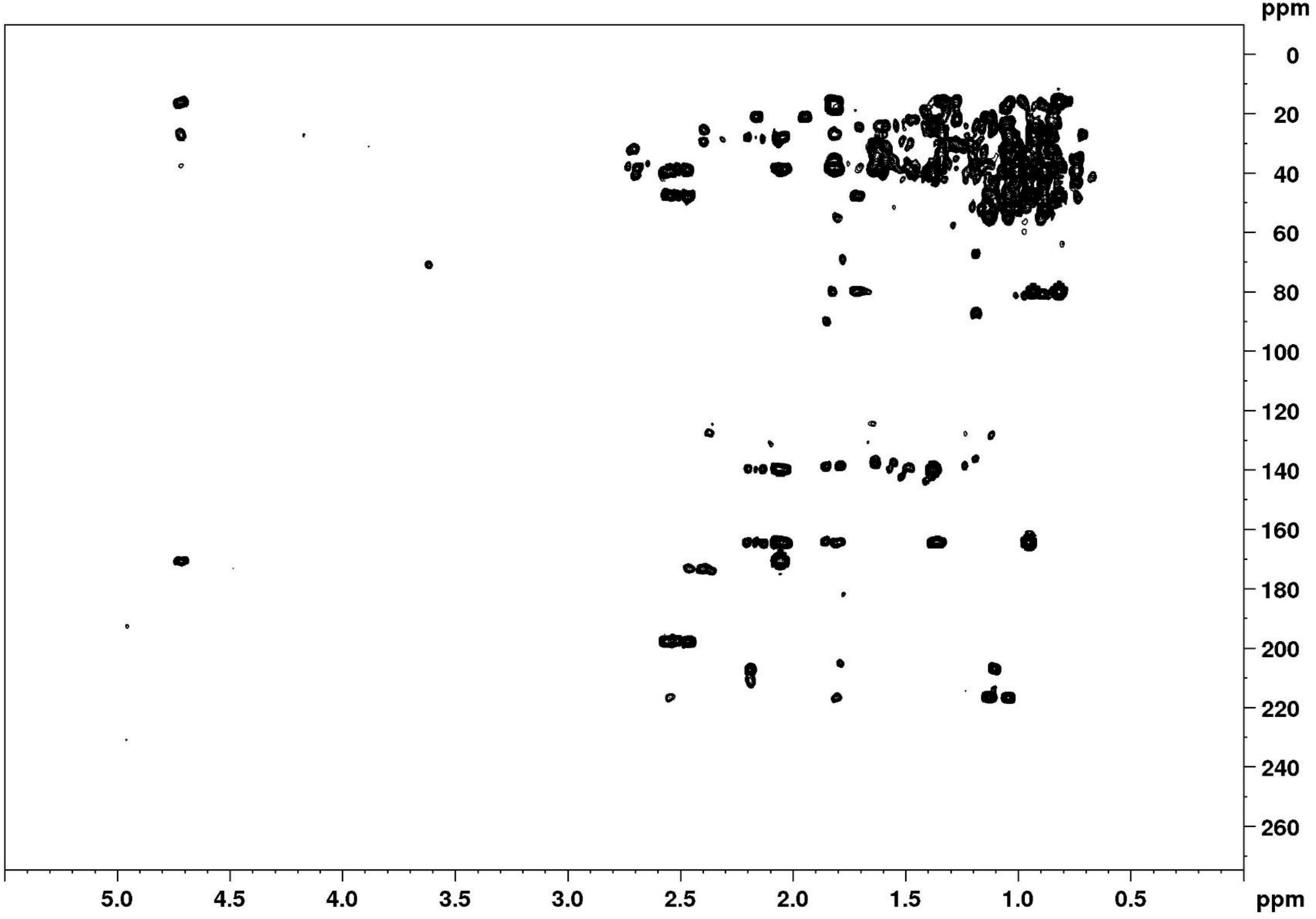


HMBC spectrum of acanthobauerenone (**3**) (600 MHz, $C_5D_5N$).

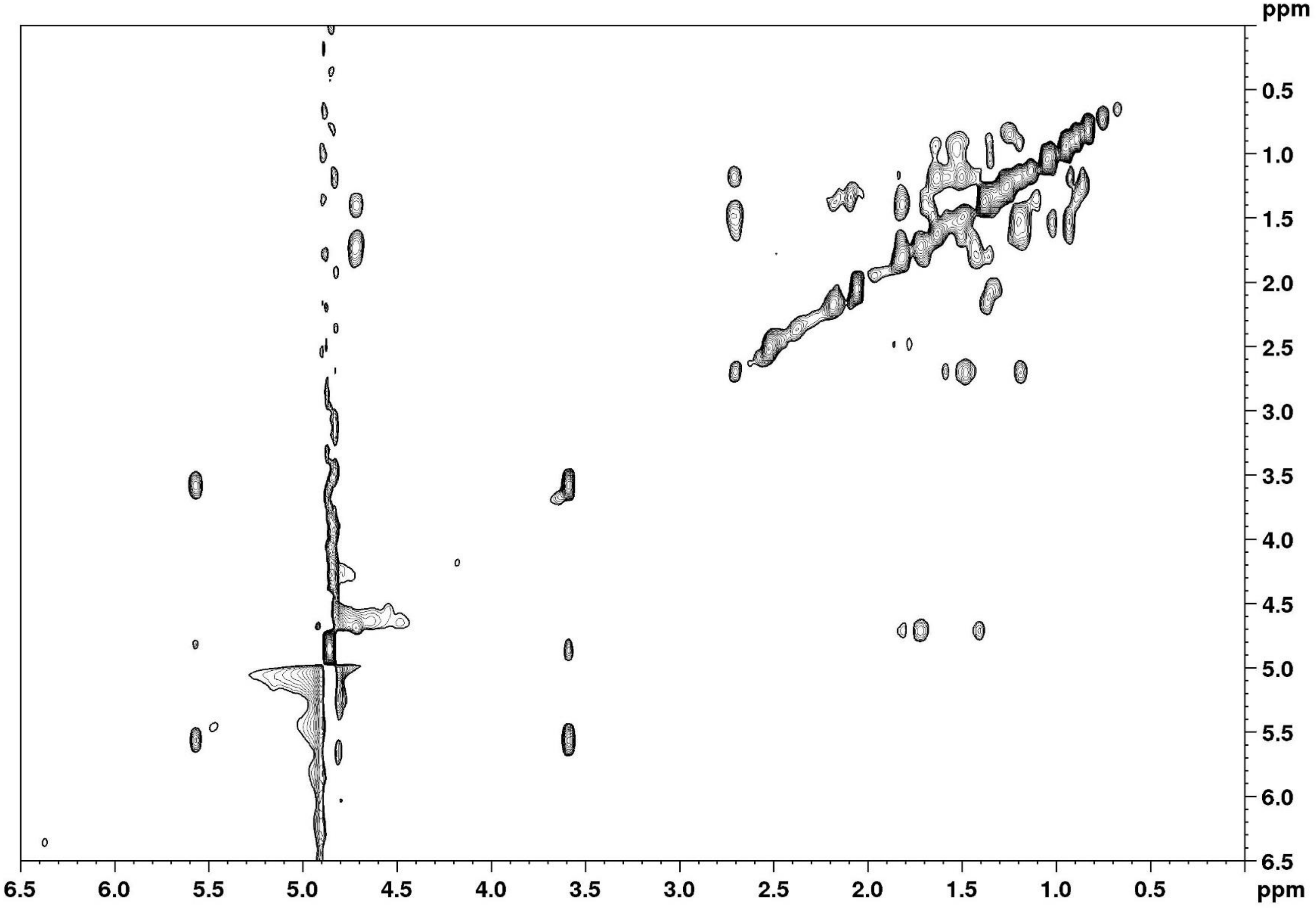


TOCSY spectrum of acanthobauerenone (**3**) (400 MHz, $C_5D_5N$).

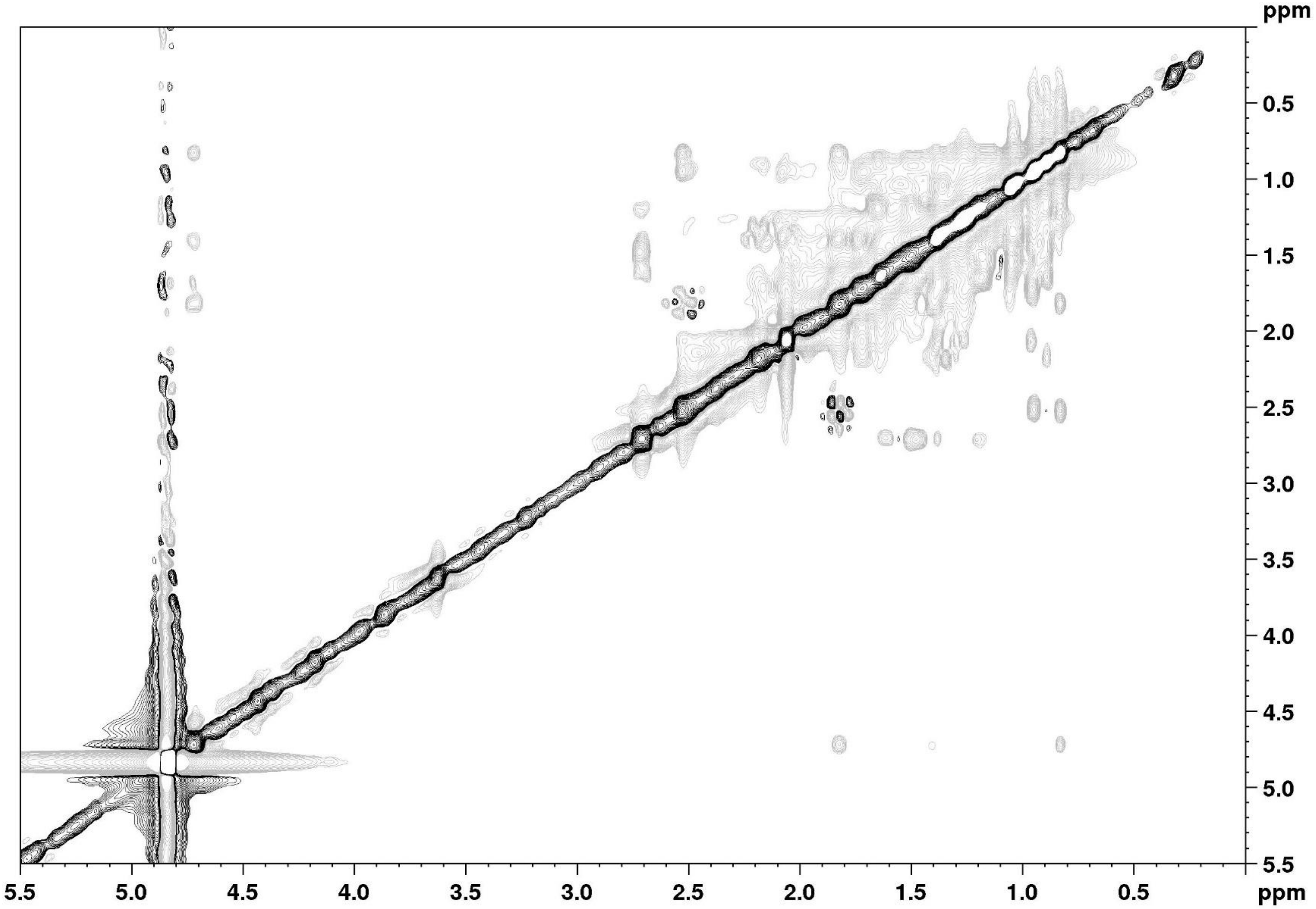


NOESY spectrum of acanthobauerenone (**3**) (400 MHz, $C_5D_5N$).

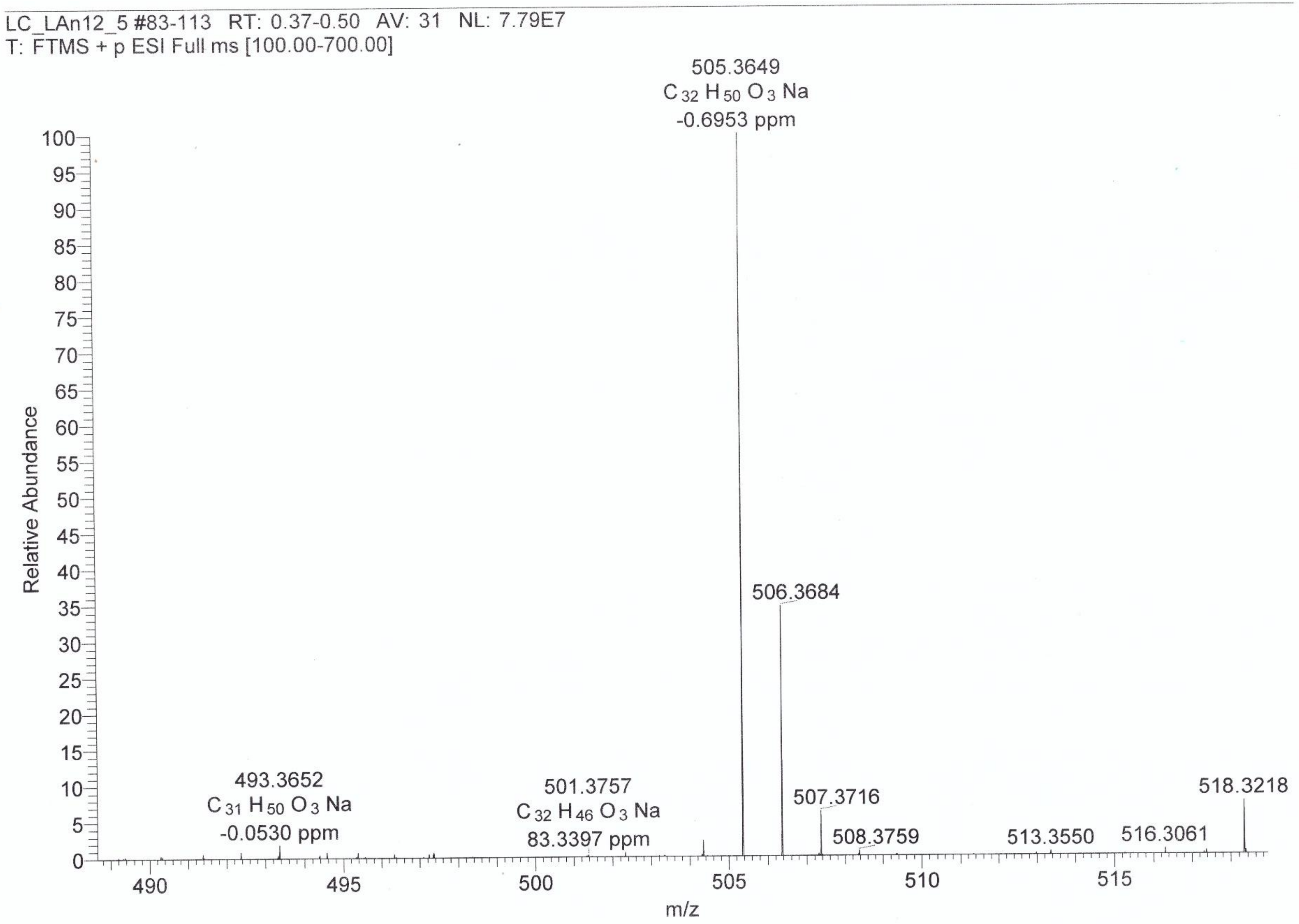


HRESIMS spectrum of acanthobauerenone (**3**).

| Position | 1 | | 2 | | 3 | |
|---|---|---|---|---|---|---|
| | $^{13}$C | $^{1}$H (*J*, Hz) | $^{13}$C | $^{1}$H (*J*, Hz) | $^{13}$C | $^{1}$H (*J*, Hz) |
| 1a<br>1b | 38.4, $CH_2$ | 1.84, ddd (12.6, 4.3, 3.6)<br>1.33, m | 35.1, $CH_2$ | 3.01, dt (13.0, 6.8)<br>1.60, m | 33.5, $CH_2$ | 1.70, m<br>1.39, m |
| 2a<br>2b | 35.4, $CH_2$ | 2.74, ddd (14.6, 14.6, 5.6)<br>2.27, m | 34.3, $CH_2$ | 2.61, m<br>2.39, m | 24.0, $CH_2$ | 1.80, m<br>1.71, m |
| 3 | 215.7, C | | 217.0, C | | 79.8, CH | 4.72, dd (11.5, 4.2) |
| 4 | 48.0, C | | 47.0, C | | 37.8, C | |
| 5 | 51.8, CH | 1.71, m | 51.3, CH | 1.68, dd (13.0, 2.0) | 47.5, CH | 1.82, dd (13.0, 5.9) |
| 6a<br>6b | 24.7, $CH_2$ | 2.01 m | 19.4, $CH_2$ | 1.60, m<br>1.34, m | 36.2, $CH_2$ | 2.55, dd (18.7, 5.9)<br>2.49, dd (18.7, 13.0) |
| 7a<br>7b | 117.8, CH | 5.55 brd (2.7) | 28.4, $CH_2$ | 2.33, m<br>2.01, ddd (12.3, 11.7, 7.5) | 197.7, C | |
| 8 | 145.6, C | | 164.4, C | | 139.6, C | |
| 9 | 48.2, CH | 2.26, m | 139.4, C | | 164.3, C | |
| 10 | 34.2, C | | 36.5, C | | 39.4, C | |
| 11a<br>11b | 17.1, $CH_2$ | 1.50, m | 197.8, C | | 22.5, $CH_2$ | 2.16, m<br>2.06, m |
| 12a<br>12b | 30.0, $CH_2$ | 1.76, m | 49.6, $CH_2$ | 2.35, ABq (18.7) | 28.2, $CH_2$ | 1.34, m |
| 13 | 37.2, C | | 40.7, C | | 38.7, C | |
| 14 | 41.5, C | | 43.4, C | | 40.7, C | |
| 15a<br>15b | 29.1, $CH_2$ | 1.56, m | 25.9, $CH_2$ | 1.62, m<br>1.28, m | 23.7, $CH_2$ | 2.70, ddd (13.0, 4.3, 2.5)<br>1.49, ddd (14.0, 12.0, 4.3) |
| 16a<br>16b | 34.6, $CH_2$ | 1.54, m | 37.4, $CH_2$ | 1.46, m<br>1.15, m | 36.7, $CH_2$ | 1.60, ddd (14.5, 13.8, 4.3)<br>1.18, m |
| 17 | 40.0, C | | 32.2, C | | 31.5, C | |
| 18 | 57.1, CH | 1.53, m | 52.3, CH | 1.36, m | 51.6, CH | 1.34, m |
| 19 | 50.3, CH | 1.62, m | 35.0, CH | 0.96, m | 34.0, CH | 1.04, m |
| 20 | 36.5, CH | 1.53, m | 31.6, CH | 1.48, m | 31.7, CH | 1.53, m |
| 21a<br>21b | 29.3, $CH_2$ | 1.47, m | 29.3, $CH_2$ | 1.10, m | 28.2, $CH_2$ | 1.80, m<br>1.41, m |
| 22a<br>22b | 39.3, $CH_2$ | 1.74, m<br>1.14, m | 32.0, $CH_2$ | 1.56, m<br>1.20, m | 32.1, $CH_2$ | 1.64, m<br>1.19, m |
| 23 | 24.5, $CH_3$ | 1.10, s | 26.8, $CH_3$ | 1.17, s | 27.0, $CH_3$ | 0.83, s |
| 24 | 21.7, $CH_3$ | 1.05, s | 21.0, $CH_3$ | 1.11, s | 16.1, $CH_3$ | 0.93, s |
| 25 | 13.1, $CH_3$ | 0.93, s | 19.5, $CH_3$ | 1.40, s | 18.3, $CH_3$ | 0.95, s |
| 26 | 23.2, $CH_3$ | 1.02, s | 21.0, $CH_3$ | 1.09, s | 21.0, $CH_3$ | 1.37, s |
| 27 | 23.3, $CH_3$ | 0.98, s | 18.2, $CH_3$ | 0.95, s | 15.7, $CH_3$ | 0.88, s |
| 28 | 33.6, $CH_3$ | 0.93, s | 37.3, $CH_3$ | 1.03, s | 38.9, $CH_3$ | 1.04, s |
| 29 | 22.3, $CH_3$ | 0.92, d (6.2) | 24.8, $CH_3$ | 0.96, d, overlap | 25.3, $CH_3$ | 1.00, d (6.4) |
| 30 | 23.8, $CH_3$ | 0.96, d (6.2) | 22.5, $CH_3$ | 0.89, d (5.8) | 22.0, $CH_3$ | 0.92, brs |
| Ac-CO | | | | | 170.5, C | |
| Ac-$CH_3$ | | | | | 20.7, $CH_3$ | 2.05, s |

NMR data in $C_5D_5N$ of acantholupenone (**1**) acanthobauerendione (**2**), and acanthobauerenone (**3**).
